# Finagle’s laws of information: lessons learnt evaluating a complex health intervention in Nigeria

**DOI:** 10.1136/bmjgh-2022-010938

**Published:** 2023-03-24

**Authors:** Sandra Alba, Callum Taylor, Margo van Gurp, Paul Balogun

**Affiliations:** 1 KIT Royal Tropical Institute, Amsterdam, Netherlands; 2 Knowledge Hub, Itad, Hove, UK; 3 Independent Consultant, Manchester, UK

**Keywords:** Epidemiology, Health systems evaluation, Public Health, Immunisation, Qualitative study

## Abstract

Evaluations cannot support evidence-informed decision making if they do not provide the information needed by decision-makers. In this article, we reflect on our own difficulties evaluating the Geo-Referenced Infrastructure and Demographic Data for Development (GRID3) approach, an intervention that provides high-resolution demographic and geographical information to support health service delivery. GRID3 was implemented in Nigeria’s northern states to support polio (2012–2019) and measles immunisation campaigns (2017–2018). Generalising from our experience we argue that Finagle’s four laws of information capture a particular set of challenges when evaluating complex interventions: the weak causal claims derived from quasi-experimental studies and secondary analyses of existing data (the information we have is not what we want); the limited external validity of counterfactual impact evaluations (the information we want is not what we need); the absence of reliable monitoring data on implementation processes (the information we need is not what we can obtain) and the overly broad scope of evaluations attempting to generate both proof of concept and evidence for upscaling (the information we can obtain costs more than we want to pay). Evaluating complex interventions requires a careful selection of methods, thorough analyses and balanced judgements. Funders, evaluators and implementers share a joint responsibility for their success.

**Summary box**

- Evaluators of complex interventions often rely on quasi-experimental study designs with weak attribution claims using existing data that are not specific enough to answer the evaluation questions (the information we have is not what we want). One way to prevent this is by early engagement of evaluators, when there is still the opportunity to influence implementation, to broaden the range of evaluative methods that can be chosen.
- Evaluation questions are sometimes guided by preferred methodologies, rather than commissioners’ information needs (the information we want is not what we need). Yet early engagement of commissioners can help evaluators better understand information needs and disentangle between questions relating to impact or questions relating to process to ensure a better alignment between evaluation questions, information needs and chosen methodologies.
- Routine monitoring systems should provide rich sources of evidence on processes of implementation, but they are typically developed with a focus on accountability demands (the information we need is not what we can obtain). Programme theories of change can help implementers articulate the main assumptions behind a programme’s success and develop informative monitoring systems.
- Conducting multiple types of evaluations at the same time can be very costly and beyond what funders typically earmark for evaluations (the information we can obtain costs more than we want to pay). Therefore, resource intensive approaches can be prioritised at an early stage, when the intervention is still on a small scale, while other less costly approaches can be used later on a broader scale.

## Introduction

As part of a wider evidence-informed policy movement, decision-makers and funders are increasingly interested in evidence of results to justify development funding.^1^ As evaluators and epidemiologists, we welcome these movements’ influence in global health. However, in our experience, there are still too many instances where the evidence produced by evaluators and researchers cannot support evidence-informed decision making because it fails to provide the information actually needed by decision makers.^2^ This is especially problematic with complex interventions that do not fit the one-cause one-effect paradigm of biomedical research^3^ and are thus less straightforward to evaluate.^4–6^ In this article, we reflect on our own experience evaluating a complex intervention in Nigeria—the Geo-Referenced Infrastructure and Demographic Data for Development approach (initially GRID and subsequently GRID3^7^)—to highlight some common challenges for evaluators and funders, and offer suggestions to improve practice.

The distinction of health interventions between simple and complex is a matter of much scholarly debate. In health, interventions such as medicines are sometimes referred to as simple because it is possible to make direct causal claims of attribution with experimental designs and statistical inference.^8^ However, it has also been argued that medicines can equally be conceptualised as complex interventions if we study aspects related to patient access (eg, adequacy, acceptability, affordability). As opposed to simple interventions, some authors distinguish between complicated and complex aspects of interventions.^9^ Complex interventions can be defined as those exhibiting multiple interacting components, many or difficult behaviours required by those delivering or receiving the intervention, several groups or organisational levels targeted by the intervention, and various and variable outcomes.^10^ Others have proposed that complex interventions are highly dependent on human agency and context^11^ and that they work by triggering context-specific and continuously evolving mechanisms.^12^ A variety of qualitative and quantitative methodological approaches are often required to build a complete and comprehensive understanding a complex intervention. The focus is generally less on attribution (direct causal links) and more on contribution to change (recognising that multiple contributing factors produce results). It has been argued that evaluations of complex interventions can at best provide ‘partial and provisional’ results given that human behaviour and context are ever-changing.^12^

GRID3 is an example of a complex intervention. It started with the aim of supporting health sector microplanning and service delivery by providing high resolution demographic estimates and geographical settlement patterns. From its initial beginnings supporting polio campaigns in northern Nigeria in 2012, GRID3 was used in several immunisation campaigns across the country.^13 14^ GRID3 can be characterised as a complex intervention, as it targets the behaviour of multiple actors and aims to trigger mechanisms in all interacting WHO health system building blocks: service delivery, human resources, medical products, governance, financing and information systems.^15^ Indeed, at its core, GRID3 is an information system providing accurate geolocated population estimates. Yet its primary aim was to support a more rational allocation of human resources and medicinal products for immunisation campaigns in order to contribute to better service delivery (and coverage) of selected vaccines. In the process, it sought to reduce both stock-outs and wastage of vaccines, thereby affecting financing. But its implementation also had implications for governance as it targeted decision-making processes at various levels of the health system (campaigns, health facilities, local health government and federal ministry, etc).

In 2019, we (see Author note) were commissioned by the Bill & Melinda Gates Foundation (BMGF) to evaluate GRID3’s use and impact in the polio and measles immunisation campaigns in Nigeria’s northern states between 2012 and 2019. Thereafter, we were tasked to provide guidance to the Clinton Health Access Initiative (CHAI) on the design and implementation of evaluations of their own use of GRID3 in health campaigns planned in Ghana (to scale-up screening sites for Sickle Cell Disease) and Kenya (to support COVID-19 outreach planning).

**Figure 1:**
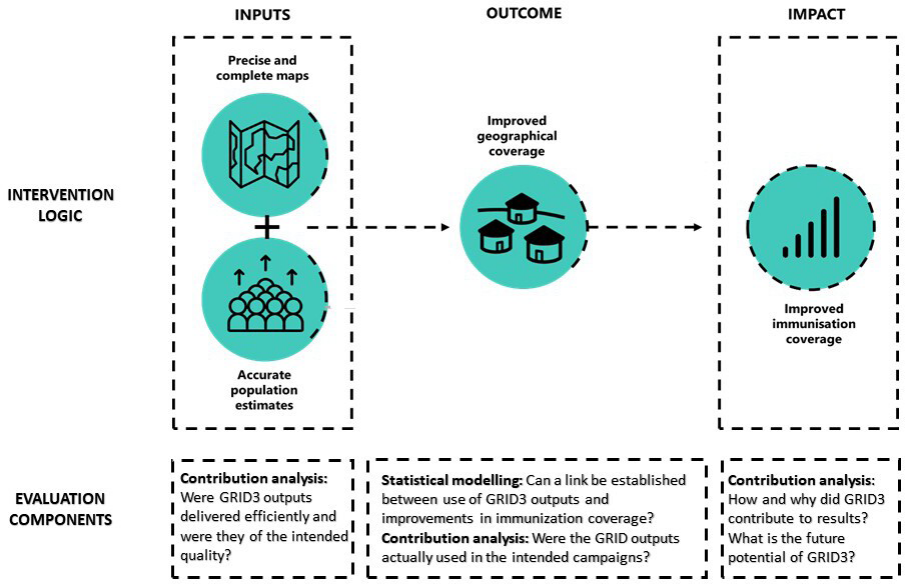
Simplified GRID3 intervention logic and evaluation components. GRID3, Geo-Referenced Infrastructure and Demographic Data for Development.

The purpose of the GRID3 evaluation in Nigeria was to provide evidence on whether GRID3 made a difference to the polio and measles vaccination campaigns, and, if so, how and why. Two studies had already established that GRID3 could lead to better geographical coverage of vaccination teams.^13 14^ The evaluation’s terms of references intended to build further on this knowledge and included questions related to the actual use of GRID3 outputs in planning campaigns; the enablers and barriers to their use; how, why and to what extent GRID3 contributed to improved campaign outcomes; the impact of GRID3; cost-effectiveness and opportunities for use in other campaigns. As shown in figure 1, we planned a mixed-methods evaluation whereby secondary analyses of existing data sources (regression modelling) would establish the impact of GRID3. This approach would provide answers to the question of *does GRID3 make a difference?* We also included qualitative evaluation methods (contribution analysis) to assess use, enablers and barriers, and to explore *how and why* GRID3 may have had such an impact. This two-stage, mixed-methods approach aimed to ensure that even if no effect of GRID3 could be discerned, we would still be able to provide insights into *why not* and thereby provide useful information for all stakeholders involved in GRID3 moving forward.

Overall, the evaluation did not provide conclusive evidence of an effect of GRID3 on campaign coverage in the two instances examined. While we saw overall positive developments in both measles and polio campaign coverage in Nigeria, we could not attribute these to the more accurate population estimates and more precise maps supported by GRID3 technology. Further details on the evaluation approach and results can be found in online supplemental file 1 and a summary of the evaluation results is presented in box 1.

10.1136/bmjgh-2022-010938.supp1 Supplementary data

Box 1 **Summary of the Geo-Referenced Infrastructure and Demographic Data for Development (GRID3) evaluation findings**

- We estimated the impact of GRID3 on polio and measles vaccination campaigns by following a two-step analytical process. Our first analytical step was to establish whether there was a change in immunisation coverage with/without and before/after the implementation of GRID3. Our second analytical step was to attempt to attribute any changes in coverage to GRID3.
- GRID3 was deployed in two phases to support polio immunisation campaigns: between 2012 and 2015 in nine northern states and between 2015 and 2019 in other parts of the country. To evaluate the use of GRID3 for the polio immunisation campaigns, we used the polio programme’s Lot Quality Assurance Survey^30^ from 2012 to 2019. GRID3 was further used during the 2017–2018 campaigns to support measles immunisation in eleven northern states. To evaluate the use of GRID3 in the measles immunisation campaigns, we used the Post Measles Campaign Coverage Surveys of 2016 and 2018.^31^
- We did not find significant differences between polio coverage estimates in areas where campaigns used GRID3 supported digital microplanning and tracking (using the Vaccine Tracking System or VTS) compared with those that did not. However, we did conclude that microplanning and tracking had the potential to contribute to fewer missed children in vaccination campaigns, since decreases in the number of missed children as per Lot Quality Assurance Surveys correlated with VTS geographical coverage indicators in the nine northern states.
- We found evidence of improved measles campaign effectiveness in states with GRID3 supported campaigns compared with states without GRID3 support as we observed a small but significant increase in vaccination coverage before and after GRID3 in GRID3 states compared with non-GRID3 states. However, we were unable to statistically link improved population estimates and improved vaccination coverage, meaning that we could not attribute improvements in immunisation coverage to the GRID3 intervention.

**Figure 2:**
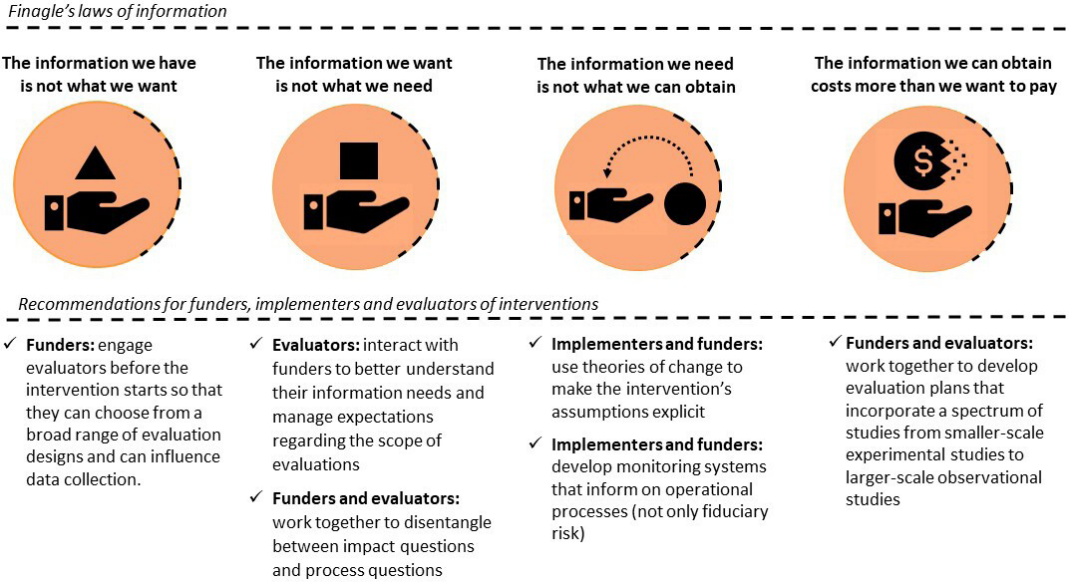
Finagle’s laws of information—recommendations for funders, implementers and evaluators of interventions.

Despite a conducive environment facilitated by BMGF staff, the evaluation was challenging, and we were not able to answer all the evaluation questions. In this article, we document our own evaluators’ perspective on this experience. Inspired by Opit^16^ and de Savigny and Binka,^17^ we refer to Finagle’s laws of information to make sense of our experience and to draw lessons for other similar evaluations. Finagle’s laws of information are among the many paradoxical theories of resistentialism which posit that ‘things are against us’.^18^ Because of their jocular undertone these theories are well suited for reflections on lessons learnt and are a useful starting point for the development of quality assurance plans.^19^ In the following sections, we present the four laws, explain their relevance to evaluations more generally, link them to our own specific experience, and draw lessons for funders, implementers, and evaluators. A summary visualisation is presented in figure 2.

## Law 1: The information we have is not what we want

A chief concern for evaluators is to choose the right methodology to answer the evaluation questions. In the ideal scenario evaluators are brought in by commissioners (usually funders of the intervention) before intervention roll-out, when there is still an opportunity to influence implementation. This maximises the range of designs that can be considered to answer the evaluation questions. In the more common scenario, however, as illustrated by our GRID3 experience, the evaluation is commissioned when the intervention has either already started or when there is no opportunity to influence implementation. This immediately eliminates many rigorous evaluation designs.^1 20–22^ As a result, evaluators often opt for quasi-experimental methodologies (eg, before-and-after studies, with or without controls) and are forced to rely on secondary analyses of existing data. Unfortunately, in this type of study design, *the information we have is not what we want* for two main reasons: quasi-experimental designs aim to support causal claims of attribution but often do not provide strong evidence;^23^ and available administrative data may not be specific enough for the evaluation questions.

Our GRID3 experience in Nigeria provides a salient example of this type of situation. Indeed, overall, our analyses did not provide conclusive evidence with regard to GRID3’s effect on campaign coverage in the two instances examined. While we saw overall positive developments with regard to geographical coverage for measles and polio immunisation, we could not attribute these to the GRID3 inputs (more accurate population estimates and more precise maps). We identified two main reasons for our inability to show an effect of GRID3. First, the existing survey data reanalysed for this evaluation proved biased for our purposes. Indeed, the sampling frame for both Post Measles Campaign Coverage Surveys and Lot Quality Assurance Surveys was based on official census enumeration areas, and therefore, exhibited precisely the limitation that GRID3 intended to address. More specifically, the sampling frame did not include the additional populations and settlements identified by GRID3 (but missed by the official census) where one expects to find most benefits in the terms of vaccination coverage (assuming that populations missed by the official census are the most remote and thus have poor access to health services). Second, the available data only enabled analyses at a high level of aggregation and were therefore statistically underpowered. Indeed, the Post Measles Campaign Coverage Surveys only provided estimates at state level, reducing our sample size to 37 states. With such a small sample size, the difference between the two groups of states compared in the analysis needed to be considerably large for it to be statistically significant—which was not the case.

Drawing on our Nigeria experience, when subsequently approached by CHAI to provide advice on the study design for GRID3 impact evaluations similar to those requested by the BMGF, we used a decision tree to systematically consider the various evaluation designs (online supplemental file 2). This decision tree is not exhaustive (many designs are omitted) and the options are not mutually exclusive (a rigorous evaluation could include both impact and theory-based evaluations). Rather, it is intended as a pragmatic tool to anticipate the needs and uses of an evaluation and to help clarify what data is available, how it might be analysed and how it fits into the purpose of a planned evaluation. This process helped CHAI staff realise that their expectations regarding evaluation designs were unrealistic, and they were able to consider alternative designs to provide useful information. We also shared our decision tree with the BMGF so that it could be used in the future planning and commissioning of evaluations.

10.1136/bmjgh-2022-010938.supp2 Supplementary data

## Law 2: The information we want is not what we need

Typically, funders commission evaluations because they need evidence of what works in order to scale-up effective interventions or replicate them in different settings. As such, funders’ needs may include information related to impact, processes or costs. Ideally, evaluation questions should follow from commissioners’ information needs and should guide the choice of evaluation design. However, in practice, this is not always the case, and evaluation questions are sometimes guided by preferred methodologies that may not be most suited to generate the information needed. In such cases, evaluators and funders alike may find themselves in a situation where *the information we want is not what we need*. As described in the introduction, the GRID3 evaluation’s terms of reference included questions regarding both process and impact. We originally planned an explanatory mixed-methods approach^24^ with a quantitative modelling exercise to answer questions relating to impact (ie, establish whether GRID3 outputs made a difference), followed by qualitative investigations to address questions regarding the process (use of GRID3 outputs, barriers and enablers). Yet, in practice, we put aside the qualitative component as it was comparatively under-resourced and hampered by COVID-19 related social distancing measures.

Reflecting on our decision to prioritise the statistical modelling part of our evaluation, we realise that we were influenced by a dominant biomedical paradigm in impact evaluations that considers counterfactual evidence of attribution from (quasi-)experimental study designs the gold standard for evaluations. Yet, it can be argued that these study designs and this type of evidence do not answer the most pressing questions for decision-makers regarding scale-up or replication of interventions. The main problem is that counterfactual evidence is not externally generalisable (it only answers the question *did this intervention work here and now*) and does not provide information about the mechanisms of change (*what conditions are needed to ensure success elsewhere?*). One way forward, as has been argued by others, is to acknowledge that human behaviour and context are ever-changing and guide today’s complex development landscape.^1 12^ Therefore, evaluators need to embrace the broader range of approaches from evaluation sciences and social sciences and accept that evaluations may at best provide ‘partial and provisional’ results.^12^ Alternative approaches include theory-based evaluations that rely mostly on qualitative research methods and focus on interventions’ processes, providing information regarding *how, why, where and for whom interventions work*. As such, their focus is generally less on attribution (direct causal links) and more on contribution to change (recognising that multiple contributing factors produce results).

Hindsight suggests that we should have engaged more with BMGF staff to better understand their information needs, while at the same time reflecting on the complexity of the GRID3 intervention and the risks involved with relying primarily on a quasi-experimental design. Critical questions include: why is an evaluation needed? how will the information be used? This can point more towards questions relating to attribution and impact (for which experimental and quasi-experimental study designs and quantitative data are more appropriate) or questions relating to contribution and process (for which theory-based evaluation and qualitative research methods are more appropriate). Having learnt our lesson, in follow-up consultations with CHAI staff, our immediate focus was on clarifying the most pressing evaluation questions and managing expectations. In this process, we also tried to expose the trade-off between secondary analysis of existing data and primary data collection: while leveraging existing data can lighten the evaluative load (in terms of time, costs and opportunity costs) it can take a toll on the evaluations’ ability to precisely address stakeholders’ information needs. The decision tree in online supplemental file 2 also supported these discussions.

## Law 3: The information we need is not what we can obtain

While external evaluations are important, routine monitoring systems should also provide rich sources of evidence on processes of implementation. However, in our experience, such monitoring systems are typically geared towards meeting accountability demands. Indeed, donors are often very concerned that funds may be misused through weak administration or corruption and are therefore keen to develop systems to minimise this risk and ensure delivery of outputs.^25^ As a consequence, monitoring systems often provide limited evidence about interventions’ operational processes on the ground. This issue is compounded by a lack of specific and locally adapted theories of change detailing hypothesised changes and the assumptions behind such changes.^26^ Yet such theories and assumptions—and accompanying data to verify them—are key to understand causal chains: Which health system building blocks are affected? Who are the actors involved at various levels and how do they interact with each other? Which outputs are expected to follow which outcomes and which impacts are expected to follow which outcomes? The unavailability of this data shows that too often *the information we need is not what we can obtain*, for both evaluators and funders.

In the GRID3 intervention such theories of change and accompanying data from routine monitoring systems were not available. GRID3 was assumed to contribute to reduced morbidity due to measles or polio in Nigeria (impact) because it increased vaccination coverage (outcome), by reaching more children who would have otherwise not been immunised (outputs). In fact, GRID3 can only trigger health system mechanisms if a number of other assumptions are met, that is, if maps and population estimates are: (1) an accurate source of health information; (2) trusted and used by health managers to procure vaccines and allocate human resources; and, (3) trusted and used by vaccinators for service delivery for underserved populations. Ideally, these assumptions would have been described in a theory of change before intervention roll-out and internal monitoring systems would have provided evidence to test them. A review of this data could then have helped identify implementation bottlenecks. In the case of the Nigeria GRID3 evaluation, it would also have helped us understand whether there was an impact of GRID3 which we could not quantify, or whether GRID3 was not implemented as planned. But, to the best of our knowledge, no such data were collected routinely for GRID3 in Nigeria.

Having learnt from this experience, in follow-up consultations with CHAI staff, our priority was to facilitate discussions around how the intervention and evaluation would work in practice and how they might be integrated. This included reflections on CHAI’s assumptions on how and why they expected GRID3 to bring results. Through these exchanges, we were able to advise on an expansion of the internal monitoring indicators.

## Law 4: The information we can obtain costs more than we want to pay

There will always be a trade-off between information and costs. One could argue that a complex intervention such as GRID3 can only truly be understood with a combination of the most rigorous counter-factual evaluation and a thorough theory-based evaluations and a complete costing study (such as cost-effectiveness or cost–benefit analyses). But conducting multiple types of evaluations at the same time can be very costly and beyond what funders are willing to earmark for evaluations. In other words, *the information we can obtain costs more than we want to pay*. This is a common challenge, one reason being that those developing and supporting new interventions appear reluctant to invest sufficiently in producing proof of concept evidence at an early stage and before scale-up. Yet understanding an intervention means moving along a ‘spectrum of evidence’^27^ from smaller-scale experimental studies to larger-scale observational studies,^28^ including various economic evaluations along the way.^29^ In between, a range of studies may be performed—with multiple iterations of experimentation—to understand the underlying processes and to refine the intervention model. Herein lie important opportunities to contain evaluations costs, as resource intensive approaches (eg, randomised controlled trials) can be prioritised at an early stage, when the intervention is still on a small scale, while other less costly approaches can be used later and on a broader scale (eg, qualitative studies).

The GRID3 evaluation in Nigeria exemplifies the difficulties of conducting evaluations with broad scopes at a rather late stage in programming. Implementation and scale-up of GRID3 had been ongoing for 7 years before the evaluation was commissioned and the intervention area had increased from 10 local government areas across 5 states in 2012 during the first polio campaigns to over 90 areas across 7 states in 2018. Two published studies linked GRID3 outputs to better geographical coverage of vaccination teams^13 14^—defined as an outcome in our intervention logic (figure 1). Yet, to the best of our knowledge, no studies had been conducted, prior to commissioning this evaluation, linking the use of GRID3 outputs to vaccination coverage impact. As a result, the terms of references of the GRID3 evaluation needed to encompass a broad range of questions, ranging from use of GRID3, enablers and barriers, contribution to change, impact, cost-effectiveness and opportunities for other campaigns. Yet, spreading the evaluation’s questions across earlier smaller scale and later larger scale studies could have not only minimised the risks of dependency on one evaluation’s results, but would have also provided the BMGF staff with evidence over those seven prior years of intervention to better understand GRID3’s process and impact and to refine the intervention.

In our follow-up consultations with CHAI staff, we were cognisant that the time for proof-of-concept evaluations had also passed. Indeed, by the time CHAI had considered commissioning evaluations, the interventions were already under-way and to varying degrees scale-up was the focus. Thankfully, by helping CHAI reflect on their theories of change and advising them on how to fine-tune their existing monitoring systems (as described in previous sections), we were able to put them in a position where they would have empirical evidence available to support larger-scale observational studies. This approach was made even more necessary and relevant given that COVID-19 restricted opportunities to carry out primary data collection. Enhancing the capacity of monitoring systems already in place to provide needed data thus made more sense both in terms of feasibility and cost.

## Conclusion

As evaluators, we long for rigorous evaluations that can inform evidence-informed practice in global health. Yet evaluating complex interventions is, by definition, a complex endeavour that requires careful choice of methods, thorough analyses and balanced judgements. A particular set of challenges can be narrowed down to Finagle’s four laws of information: the weak causal claims derived from quasi-experimental studies and secondary analyses of existing data (the information we have is not what we want); the limited external validity of counterfactual impact evaluations (the information we want is not what we need); the absence of reliable monitoring data on implementation processes (the information we need is not what we can obtain); and the overly broad scope of evaluations attempting to generate both proof of concept and evidence for upscaling (the information we can obtain costs more than we want to pay). Evaluation failure following from these challenges can be mitigated if: (1) funders engage evaluators before the start of an evaluation to enable evaluators to influence study design and data collection; (2) evaluators interact with funders to understand their information needs, manage expectations regarding the scope of evaluations, and disentangle between impact and process questions; (3) implementers and funders use theories of change to make the intervention’s assumptions explicit and to develop monitoring systems that inform on operational processes (not only fiduciary risk) and (4) funders and evaluators work together to develop evaluation plans that incorporate a spectrum of studies from smaller-scale experimental studies to larger-scale observational studies. In other words, Fingale’s information laws should serve as a reminder that commissioners, evaluators and implementers share a joint responsibility for the success of an evaluation.

## Data Availability

Data may be obtained from a third party and are not publicly available.

## References

[1] Stern E, Stame N, Mayne J, et al. Broadening the range of designs and methods for impact evaluations [internet] [Institute for Development Studies]. 2012. Available: http://repository.fteval.at/id/eprint/126

[2] Oliver K, Lorenc T, Innvær S. New directions in evidence-based policy research: a critical analysis of the literature. Health Res Policy Syst 2014;12:34. 10.1186/1478-4505-12-34 25023520 PMC4107868

[3] Petticrew M. When are complex interventions “ complex ”? when are simple interventions “ simple ”? Eur J Public Health 2011;21:397–8. 10.1093/eurpub/ckr084 21771736

[4] Skivington K, Matthews L, Simpson SA, et al. A new framework for developing and evaluating complex interventions: update of medical Research Council guidance. BMJ 2021;374:n2061. 10.1136/bmj.n2061 34593508 PMC8482308

[5] Norris SL, Rehfuess EA, Smith H, et al. Complex health interventions in complex systems: improving the process and methods for evidence-informed health decisions. BMJ Glob Health 2019;4(Suppl 1):e000963. 10.1136/bmjgh-2018-000963 PMC6350736 30775018

[6] Minary L, Trompette J, Kivits J, et al. Which design to evaluate complex interventions? toward a methodological framework through a systematic review. BMC Med Res Methodol 2019;19:92. 10.1186/s12874-019-0736-6 31064323 PMC6505260

[7] GRID3 [Internet]. GRID3. 2021. Available: https://grid3.org/

[8] Chapter 17: intervention complexity [internet]. 2022. Available: https://training.cochrane.org/handbook/current/chapter-17

[9] Rogers PJ. Using programme theory to evaluate complicated and complex aspects of interventions. Evaluation 2008;14:29–48. 10.1177/1356389007084674

[10] Craig P, Dieppe P, Macintyre S, et al. Developing and evaluating complex interventions: the new medical Research Council guidance. BMJ 2008;337:a1655. 10.1136/bmj.a1655 18824488 PMC2769032

[11] Abimbola S, Baatiema L, Bigdeli M. The impacts of decentralization on health system equity, efficiency and resilience: a realist synthesis of the evidence. Health Policy Plan 2019;34:605–17. 10.1093/heapol/czz055 31378811 PMC6794566

[12] Abimbola S. Making sense of the complexity of decentralised governance; Comment on “ the effects of health sector fiscal decentralisation on availability, accessibility, and utilisation of healthcare services: a panel data analysis. ” Int J Health Policy Manag 2023:1–3. 10.34172/ijhpm.2022.7449 PMC10125076 37579443

[13] Touray K, Mkanda P, Tegegn SG, et al. Tracking vaccination teams during polio campaigns in northern Nigeria by use of geographic information system technology: 2013-2015. J Infect Dis 2016;213 Suppl 3(Suppl 3):S67–72. 10.1093/infdis/jiv493 26609004 PMC4818548

[14] Barau I, Zubairu M, Mwanza MN, et al. Improving polio vaccination coverage in Nigeria through the use of geographic information system technology. J Infect Dis 2014;210 Suppl 1:S102–10. 10.1093/infdis/jiu010 25316823

[15] de SD, Adam T, Research A for HP and S, Organization WH. Systems thinking for health systems strengthening [internet] [World Health Organization]. 2009. Available: https://apps.who.int/iris/handle/10665/44204

[16] Opit LJ. How should information on health care be generated and used? world health forum 1987 84 409-438 [Internet]. 1987. Available: https://apps.who.int/iris/handle/10665/51712

[17] Savigny DD, Binka F. Monitoring future impact on malaria burden in sub-saharan africa *internet+. the intolerable burden of malaria II: what’s new, what’s needed: supplement to volume 71(2) of the american journal of tropical medicine and hygiene. American Society of Tropical Medicine and Hygiene 2004. 10.4269/ajtmh.2004.71.224 15331841

[18] Resistentialism. Wikipedia [internet]. 2022. Available: https://en.wikipedia.org/w/index.php?title=Resistentialism&oldid=1098387570

[19] Alba S, Straetemans M. Whatever can go wrong, need not go wrong: open quality approach for epidemiology. Emerg Themes Epidemiol 2021;18:8. 10.1186/s12982-021-00098-0 34273982 PMC8285770

[20] World Bank. Impact evaluation in practice - second edition [internet]. 2021. Available: https://www.worldbank.org/en/programs/sief-trust-fund/publication/impact-evaluation-in-practice

[21] Home | 3ie [internet]. 2021. Available: https://www.3ieimpact.org/

[22] BetterEvaluation [Internet]. BetterEvaluation. 2021. Available: https://www.betterevaluation.org/en

[23] Murad MH, Asi N, Alsawas M, et al. New evidence pyramid. Evid Based Med 2016;21:125–7. 10.1136/ebmed-2016-110401 27339128 PMC4975798

[24] Bamberger M. Introduction to mixed methods in impact evaluation: 42. n.d.

[25] Department for International Development. A DFID pratice paper - managing fiduciary risk when providing financial aid [internet]. 2009. Available: https://europa.eu/capacity4dev/file/10207/download?token=v3oOtbsk

[26] Taplin D, Clark H. Theory of change basics - A primer on theory of change. March 2012.

[27] Peters DH, Adam T, Alonge O, et al. Implementation research: what it is and how to do it. BMJ 2013;347:f6753. 10.1136/bmj.f6753 24259324

[28] Banerjee A, Banerji R, Berry J, et al. From proof of concept to scalable policies: challenges and solutions, with an application. Journal of Economic Perspectives 2017;31:73–102. 10.1257/jep.31.4.73

[29] Turner HC, Archer RA, Downey LE, et al. An introduction to the main types of economic evaluations used for informing priority setting and resource allocation in healthcare: key features, uses, and limitations. Front Public Health 2021;9:722927. 10.3389/fpubh.2021.722927 Available: https://www.frontiersin.org/articles/10.3389/fpubh.2021.722927 34513790 PMC8424074

[30] Brown AE, Okayasu H, Nzioki MM, et al. Lot quality assurance sampling to monitor supplemental immunization activity quality: an essential tool for improving performance in polio endemic countries. J Infect Dis 2014;210 Suppl 1:S333–40. 10.1093/infdis/jit816 25316852

[31] Wagai JN, Rhoda D, Prier M, et al. Implementing WHO guidance on conducting and analysing vaccination coverage cluster surveys: two examples from nigeria. PLOS ONE 2021;16:e0247415. 10.1371/journal.pone.0247415 33635913 PMC7909665

