# Supplementary material for: Finagle’s laws of information: lessons learnt evaluating a complex health intervention in Nigeria
Source: BMJ Glob Health. 2023 Mar 24;8(3):e010938. doi: 10.1136/bmjgh-2022-010938 (PMC10040038; doi:10.1136/bmjgh-2022-010938)
Supplement: Supplementary data [file bmjgh-2022-010938supp001.pdf]

Evaluation Progress Report

Date: September 2020

Authors: Sandra Alba, Margo van Gurp

Submitted by Itad in association with Royal Tropical Institute (KIT)

Disclaimer

The views expressed in this report are those of the evaluators. They do not represent those of Itad, KIT, BMGF or of any of the individuals and organisations referred to in the report.

‘Itad’ and the tri-colour triangles icon are a registered trademark of ITAD Limited.

Contents

List of acronyms 3

Introduction 4

Purpose 4

Evaluation question 4

Analytical method 5

Data sources 7

Key findings 8

Effect of GRID on Polio SIA immunization coverage 8

Effect of GRID on Measles SIA immunisation coverage 10

Discussion 12

ANNEX A: Statistical models 15

ANNEX B: TECHNICAL NOTES ON LQAS AND LINK BETWEEN LQAS and VTS 18

ANNEX C: TECHNICAL NOTES ON PMCCS 19

ANNEX D: LQAS coverage estimates by LGA and by year 20

ANNEX E: Outputs for Model 1 and Model 2 (Polio) 29

ANNEX F: Outputs for Model 4a and b, and Model 5a and b (Measles) 31

ANNEX G: 2016 and 2019 GRID population estimates 33

List of acronyms

DiD	Difference-in-Difference
DRC	Democratic Republic of the Congo
FCT	Federal Capital Territory
GPS	Global Positioning System
GRID/GRID3	Geo-Referenced Infrastructure and Demographic Data for Development
LGA	Local Government Authority
LQAS	Lot Quality Assurance Sampling
MVC	Measles Vaccine Campaign
NPHCDA	National Primary Health Care Development Agency
PMCSS	Post Measles Campaign Coverage Survey
SIA	Supplementary Immunization Activity
VTS	Vaccination Tracking System

Alba S, *et al. BMJ Global Health* 2023; 8:e010938. doi: 10.1136/bmjgh-2022-010938

Introduction

Purpose

The purpose of this workstream 2 is to provide evidence on whether GRID *made a difference* in selected campaigns and, if so, *how and why*. This workstream consists of 6 evaluation questions, which relate to: the actual use of GRID inputs; the enablers and barriers; how, why and to what extent GRID contributed to improved campaign outcomes; impact of GRID; cost-effectiveness; and opportunities for use in other campaigns. We followed a mixed methods evaluation approach whereby the impact of GRID was modelled statistically (to answer the question *'does GRID make a difference*') whereas we planned to use qualitative evaluation methods to assess use, enablers and barriers, as well as other reasons relating to *'how and why*' GRID may have made such an impact. The evaluation of cost-effectiveness and opportunities for use in other campaigns was planned as contingent to finding an effect of GRID.

Due to Covid-19 travel restrictions we opted for an explanatory approach whereby the first stage consisted of the quantitative impact evaluation of GRID made a difference, to be followed in a second stage of investigations regarding use, enablers and barriers and other reasons relating to *'how and why*' of the impact, with qualitative research methods. In terms of the theory of change in Figure 1, the quantitative modelling attempts to link GRID inputs with GRID impact (more specifically the indicator 'increased achievement of disease campaign outcomes'), whereas the qualitative research questions will examine the different steps in the causal pathway between inputs and impact. This two-stage mixed-methods set-up ensures that even if no effect of GRID can be discerned, we can provide insights into *'why not*' and thereby provide useful information for all stakeholders involved in GRID moving forward.

Evaluation question

Within workstream 2, the effect of GRID on campaign outcomes is explored by Evaluation Question 6: In each of Nigeria and DRC, what has been the impact of GRID on intervention coverage, reach, equity, cost, reduction in wastage, or other campaign outcomes? Given that data available to us for this evaluation question we were only able to assess the effect of GRID on geographic coverage (intervention coverage) and immunisation coverage (other campaign outcomes). Stakeholders' perceived effect on reach, equity, cost and reduction in wastage will be assessed qualitatively as part of the related Evaluation Question 5: In each of Nigeria and the Democratic Republic of the Congo (DRC), how, why and to what extent did use of GRID in planning contribute to achieving the intended primary campaign outcomes?

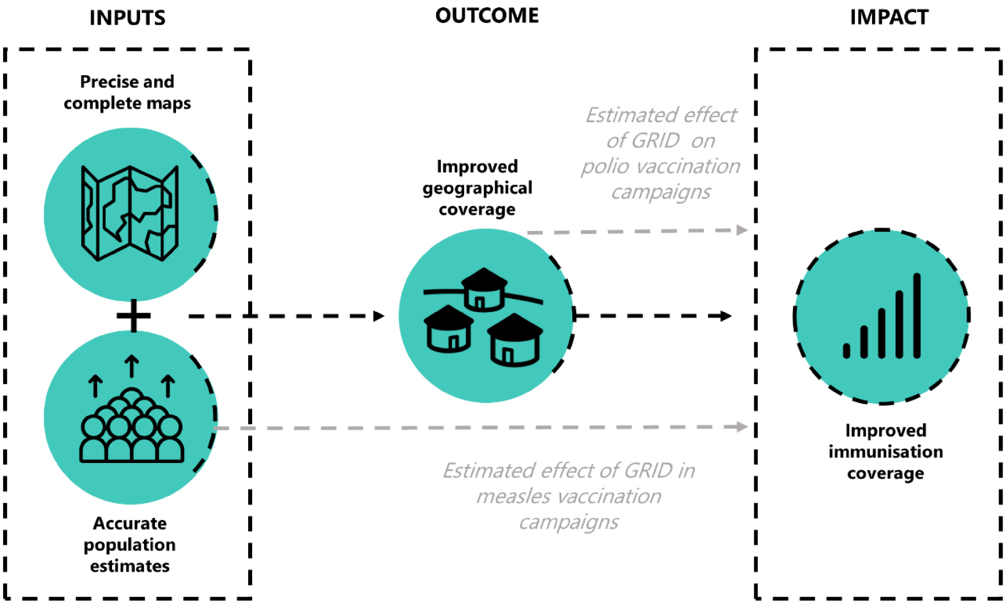

Figure 1 - Conceptual framework guiding analyses for Evaluation Question 6

Analytical method

Our analytical approach to attribute effects of GRID on improved immunization coverage was guided by data availability and the conceptual framework as visualized in Figure 1 above. This conceptual framework focuses on three levels of the program's theory of change which we were able to operationalize quantitatively as follows for the statistical modelling:

- Input: changes in population estimates before and after GRID for measles Supplementary Immunization Activity (SIA)
- Outcome: geographical coverage of polio SIA immunization teams as denoted by a) the proportion of settlements visited and b) the average area covered by settlement as a percentage of the total settlement area)
- Impact: post-campaign immunization coverage as denoted by a) proportion of children vaccinated (measles) and b) number of children missed per campaign (polio)

We estimated the impact of GRID on polio and measles vaccination campaigns by following a two-step analytical process. Our first analytical step was to establish whether there was a change in immunization coverage with/without and before/after the implementation of – or 'exposure' to - GRID. Our second analytical step was to attempt to attribute any changes in coverage to GRID. These are described in further detail below.

Change in immunization coverage with/without and before/after GRID - For polio we analysed SIA immunization coverage data from surveys conducted between 2012 and 2019. Exposure to GRID intervention was defined in two ways, which reflect both the historical evolution of GRID in Nigeria as well as the two components of the GRID intervention, as visualized above: a) mapping (comprehensive settlement locations, infrastructure mapping and harmonized subnational boundaries) and b) population estimates (high resolution population estimates and high-quality geo-references census). GRID3's fully fledged implementation in Nigeria is commonly set in 2019, when WorldPop created the first set of 'bottom-up' population estimates for the whole country based on two main sources of data[1]: micro-census surveys and

[1] https://www.pnas.org/content/115/14/3529.full

geographical covariates derived by satellite imagery[2]. In order to produce GRID3 Nigeria population estimates, Worldpop obtained both sources of data from polio vaccination tracking system (VTS[3]), which has been tracking a selection of polio vaccination campaigns in Nigeria since 2012. More specifically, during the 2012-2019 time period which is the focus of evaluation, two phases can be distinguished reflecting the timepoints in which the two elements of GRID were *de facto* introduced to support polio vaccination campaigns. These were used for the following operational definition of GRID exposure for polio vaccination campaigns (Figure 2):

- Phase 1 (2012-2015) denotes exposure to the *mapping component only* of GRID: a selection of campaigns in the 9 northern states of Nigeria were supported by: 1) digital microplans based on satellite imagery were made at ward level to support field teams and 2) field teams were geographically tracked using the VTS
- Phase 2 (2016-2019) denotes exposure to *both mapping and demographic component* of GRID: 1) microplanning with digital maps and VTS geographical tracking was up-scaled to cover a selection of campaigns in other parts of the country and 2) microplans included updated demographic information from 'bottom-up' population models (Oak Ridge National Laboratory models from 2016-2018 and Worldpop GRID models from 2018 onwards).

Defining exposure to GRID for the measles vaccination campaigns was more straightforward and consist of exposure to *both mapping and demographic component*: GRID was used during the 2017/2018 campaigns to support campaigns in 11 northern states (Bauchi, Gombe, Jigawa, Kano, Kaduna, Katsina, Kebbi, Plateau, Sokoto, Yobe and Zamfara) and the Federal Capital Territory (FCT) (Figure 3). Microplans were made at ward level based on updated maps provided by the polio program as well as updated population estimates from the 2016 Oak Ridge National Laboratory models (i.e. exposure to both mapping and demographic component of GRID). GRID was partially implemented in Adamawa, Borno and Niger but the purpose of the analysis, only the 11 Northern states in which GRID was fully implemented were defined as 'GRID states'.

Attribution to GRID – We followed two separate but related approaches to assess attribution for the polio and measles vaccination campaigns. These were dependent on data availability and are visualized with grey arrows in Figure 1 above. For polio vaccination campaigns, we assessed whether change in immunization coverage were related to changes in in the immunization teams' geographical coverage. For the measles vaccination campaigns, we assessed whether the change in immunization coverage was associated with changes in population estimates for two subgroups:

- Post campaign immunization coverage: the percentage of children who were immunized during the vaccination campaign regardless of prior vaccination status. This provides inside into the overall coverage of the immunization campaign which aims to reach all children between the ages of 9 and 59 months
- Zero-dose coverage: the percentage of children who were immunized during the campaign who had never received the vaccination prior to the campaign. This provides inside into the ability of the campaign to reach children who may not have been reached otherwise.

Details of the statistical models fitted to estimate these associations can be found in Annex A

2 Note: Worldpop GRID 'bottom-up approach differs from the general 'top-down' WorldPop Global approach used to produce internationally comparable population estimates (https://wopr.worldpop.org/?/Population)

3 http://vts.eocng.org/

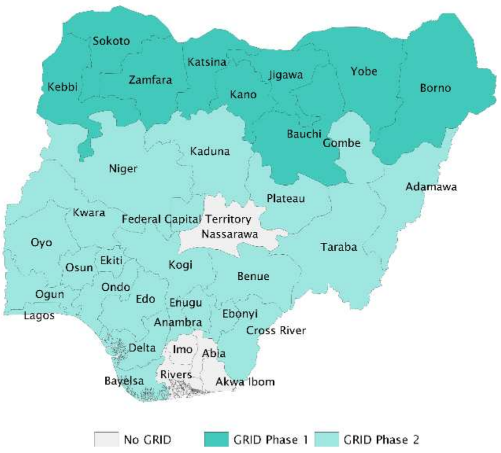

Figure 2 - GRID implementation states for the Polio SIA

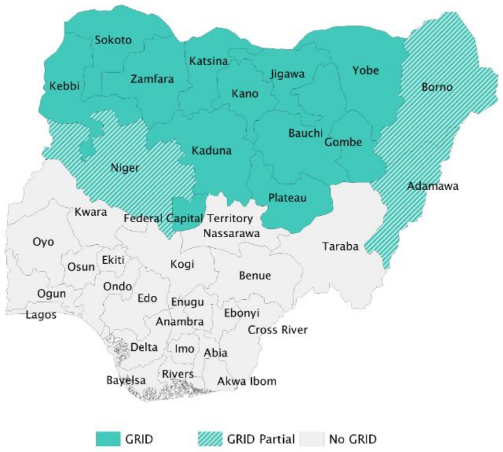

Figure 3 - GRID implementation states for the 2017-18 Measles SIA

Data sources

Analyses were all done on existing data sources provided by the National Primary Health Care Development Agency (NPHCDA), Novel-T or derived from the VTS. Table 1 provides an overview of all datasets used and corresponding data sources.

Table 1 - Overview of datasets and data sources for measles analyses

Dataset	Source
Nigeria 2006 census projected population estimates for 2012 - 2020	NPHCDA
Population estimates for 10* Northern states in 2016 (Oak Ridge National Laboratories model)	Novel-T
Population estimates for all Nigeria states 2019 (WorldPop GRID model)	vts.eocng.org
List of VTS tracked SIA, teams deployment and geographic coverage of each tracked polio SIA	vts.eocng.org
MCV Microplans with population estimates 2015 & 2017 on state level	Novel-T
Polio programme Lot Quality Assurance Surveys (LQAS) 2012-2019	NPHCDA
Post Measles Campaign Coverage Survey (PMCCS) 2016 and 2018	NPHCDA

* Estimates included Kaduna but not Plateau state, explaining the difference between the 9 states included in the polio intervention group and the 11 states included in the measles intervention group.

Technical notes on the use of LQAS and PMCCS for this evaluation can be found in Annex B and Annex C. The main strength of these two datasets is that they provide timely independent estimates of immunization coverage achieved by the two types of campaigns that are being evaluated (Polio SIA and MCV). However, there are two main limitations:

1. Statistical power: The PMCCS only provides estimates at state level. From the 37 states in total, 11 received the intervention 'GRID' and 26 did not. The chances of finding a

statistical association are heavily dependent on the sample size. If the sample size is small the difference between the study groups (i.e., states that have received GRID support versus states that have not) needs to be considerable for it to be statistically significant. The analysis may be 'statistically under-powered': they are not able to detect a statistically significant difference if the difference is small, even if one exists.

2. Epidemiological power: The sampling frame for both surveys is based on the census enumeration areas, which does not include the areas added in the microplans by the improved mapping component of GRID – where one expects to find most of the benefits of the intervention in term of vaccination coverage. In that sense the analyses conducted on them can be defined as 'epidemiologically under-powered': they are not able to capture the entirety of an effect even if there is one because of the limitations in sampling frame's geographical coverage[4].

Key findings

Effect of GRID on Polio SIA immunization coverage

At impact-level, no effect of digital microplanning and tracking can be discerned on the polio SIA LQAS immunization coverage estimates if we compare trends in Local Government Authorities (LGAs) where campaigns were tracked in the VTS compared to those that were not. Figure 4 shows the estimated number of children missed by the Polio SIA from 2012 to 2019 according to the LQAS (see also Annex D for maps of LQAS results per LGA per year and list of VTS tracked SIAs). Figure 4 shows that the number of children missed by the polio SIA according to LQAS decreased substantially in the first phase of GRID implementation (2012-2015) when the digital microplanning and tracking of teams (by means of the VTS) was introduced in the 9 northern states. The drop in the number of missed children can be seen as much in the LGAs with 'regular' campaigns and LGAs with the intervention, but there is evidence that the drop was slightly steeper in the non-GRID states compared to GRID: 0.07 extra children missed per month in the tracked campaigns compared to regular campaigns, i.e. 1.8% per year, based on a n=60 denominator (See Annex D for details on LQAS and Annex E Model 2 for). While this provides evidence against impact of GRID, it is important to realise that the magnitude is small and could be due to the fact that the GRID states are weaker performing states in general, which is why they were selected for the intervention in the first place. In the second phase of GRID implementation microplanning, geographical tracking and improved population estimates were introduced sporadically in southern states, while the northern states largely returned to 'regular' campaigns. In this period, we observe no further decreases in the number of missed children, no differences between LGAs with regular campaigns or those with the intervention. This is confirmed statistically by interrupted times series analyses fitted to estimate change in immunization coverage with/without and before/after GRID (See Annex E Model 2 for details).

[4] Aware of this limitation of the LQAS surveys, WHO Nigeria and Novel-T collaborated on an initiative to include the possibility of sampling 'new' clusters (not in census list of enumeration areas but identified following the VTS digital mapping). However, this was only piloted in 2016 in Kano, whereas our evaluation uses data from 2012.

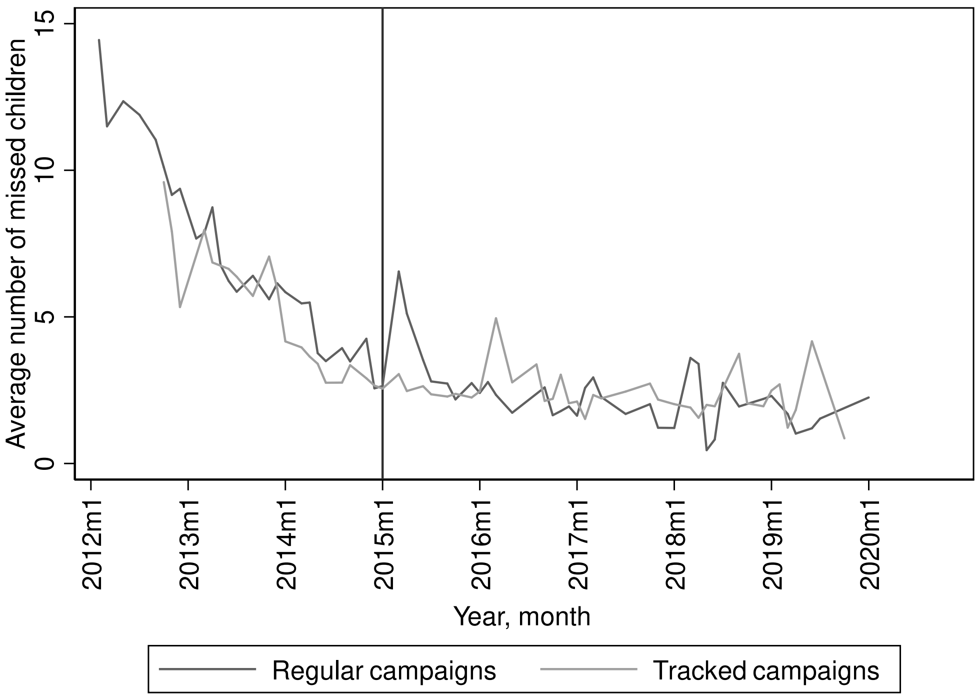

Figure 4 - Estimated average number of children missed by Polio SIA (according to LQAS) in Phase 1 (2012-2015) and Phase 2 (2016-2019) of GRID implementation

However, if we focus on the causal steps between improved geocoverage (outcome) and improved vaccination coverage (impact), we see that that microplanning and tracking does have the potential to contribute to fewer missed children, since decreases in the number of missed children correlates with geographical coverage indicators in the 9 northern states. We focus on the nine northern states for these analyses as this is where the VTS tracking was use most intensively. Figure 5 and Figure 6 visualize trends in geographical coverage available from the VTS by state for the nine northern states. There are two indicators of interest: the proportion out of all settlements that is visited by vaccinators (proportion of visited settlements, Figure 5) and the average proportional area of settlements that is covered by vaccinators as measured by phones' Global Positioning System (GPS) tracks (geocoverage, Figure 6). Analyzing the trends in these two indicators, we see a general pattern whereby the proportion of visited settlements and the geocoverage of visited settlements by LGA increased slightly over time from the start of tracking (see Annex E Model 2 (a) for details). These trends on their own testify for the usefulness of the VTS tracking as a monitoring tool and its use in practice to improve campaign efficiency. Regressed against LQAS estimates of campaign coverage they provide information on whether that tracking has the potential to contribute to positive campaign coverage outcomes. Model 2(b) (see Annex A for details of model and Annex E for outputs) shows that only geocoverage of visited settlements is significantly associated with decreases in the number of missed children. It is unclear why only this coverage variable, and not the other one (proportion of settlements visited) is associated with decreases in missed children. These analyses cannot factor in any counterfactual comparisons (since there is not data on coverage in areas without VTS tracking), the effect of geocoverage remains after correcting for the variable 'year', suggesting that effects of geocoverage exist independently from (and in addition to) the secular effect of time which was observed in both regular and tracked campaigns (Model 1). Therefore, while these analyses are not robust enough to attribute any effects to VTS tracking, they so support the hypothesis that VTS tracking contributed to positive campaign outcomes, and that our inability to quantify this effect statistically may be due to limitations in power.

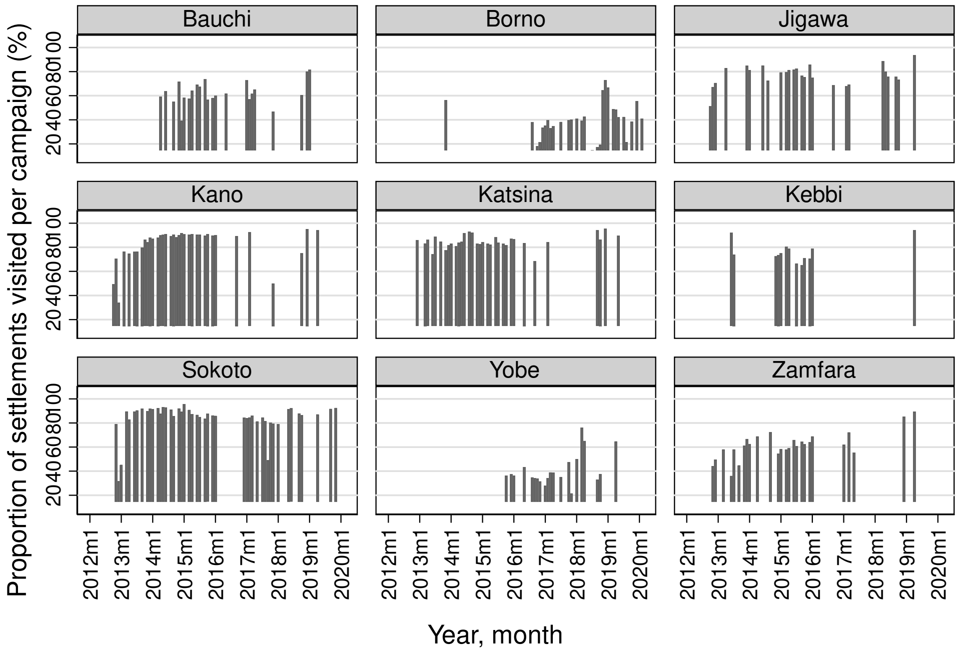

Figure 5 - Proportion of settlements visited by month and by state in nine northern states (source: VTS)

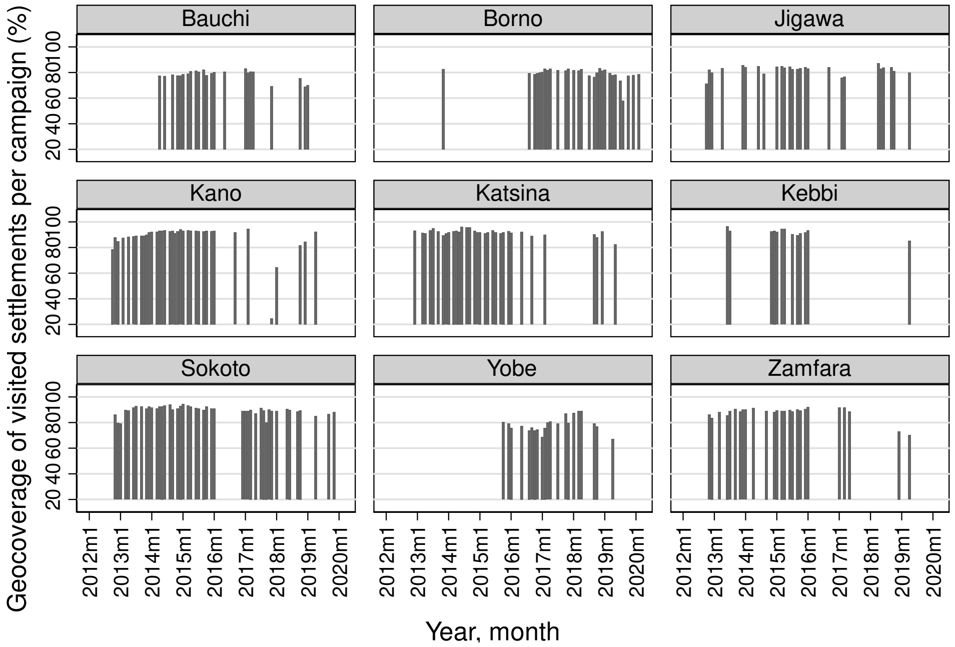

Figure 6 - Geocoverage of visited settlements by month and by state in nine northern states (source: VTS)

Effect of GRID on Measles SIA immunisation coverage

At impact-level, there is evidence of improved measles campaign effectiveness in states with GRID supported campaigns compared to states without GRID support, since we observe a small but significant improvement in vaccination coverage before and after GRID in GRID-

states compared to non-GRID states. For this analysis we used 2016 PMCCS data as the baseline as it provides us with the campaign effectiveness in the absence of GRID. The 2018 PMCCS data serves as the endline since GRID was implemented during the 2018 Measles Vaccine Campaign (MVC) in 11 states. The 26 states which were not supported by GRID are treated as the counterfactual. As such, this analysis resembles a classic controlled before-and-after study with a difference-in-difference (DiD) estimate of effect. Children living in GRID states were less likely to be vaccinated during the 2016 MVC (when none of the states received GRID support) as compared to children who were living in non-GRID states. However, while overall post-campaign immunization coverage increased slightly between the 2016 and 2018 MVC in GRID states the post campaign coverage in the other states remained stable (Figure 7). In fact, Model 3a suggests a small but significant improvement in the odds of children under 5 being vaccinated after the campaign before and after GRID implementation in GRID states as compared to non-GRID states, corresponding to a DiD effect of 3.9% (meaning coverage increased 3.9 percent-points more in the GRID states compared to non-GRID states). This same effect cannot be replicated on first time vaccination across the country (Figure 8) as can be seen from fitting Model 3b (See Annex A for details of the model and Annex F for the model outputs).

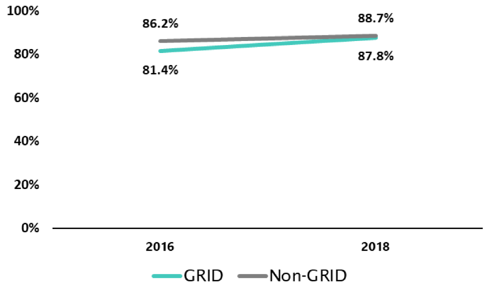

Figure 7 - The percentage of children aged 9-59 months who were immunised during the 2016 and 2018 MVC in GRID and non-GRID states

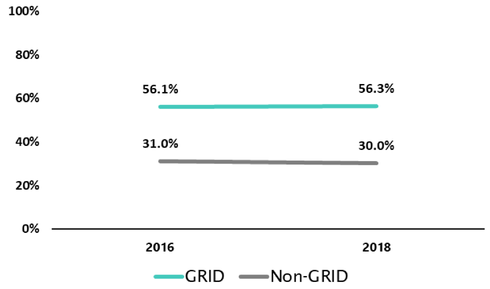

Figure 8 - The percentage of children aged 9-59 months who were immunised for the first time during the 2016 and 2018 MVC in GRID and non-GRID states

However, we are unable to statistically link improved population estimates (outputs) and improved vaccination coverage (impact), meaning that we cannot attribute improvements in immunization coverage to the GRID intervention. For these analyses we focused on the 11 GRID states and for each of them we calculated two values: the change in the target population (x-variable) and the change in average state-level vaccination coverage (y-variable), and then correlated them with a linear regression model (as below described in Annex A Model 4). Figure 9 shows the difference in the estimated target population of children aged 9-59 months between the 2016 and 2018 MVC (x). Since southern and Eastern states (where GRID was not implemented) used census estimates for campaign planning, the difference between the 2016 and 2018 target population in these states reflects the state level annual growth rate. In the 11 states that implemented GRID prior to the 2018 MVC a considerable change in target population estimates – both negative and positive - can be observed. Figure 10 shows the difference between the 2016 post-campaign immunization coverage estimates and the 2018 post-campaign immunization coverage estimates (y): four states aside, the 2018 MVC achieved higher immunization coverage as compared to the 2016 MVC across the country. We calculated the difference between the 2016 and 2018 post MVC campaign coverage for each state and found an average increase in immunization coverage of 9.0 percentage points in GRID states compared to an increase 8.2 percentage points in non-GRID states. Figure 11 also shows 2016 post-campaign immunization coverage (y) but for children who were not previously vaccinated (zero-dose) and unfortunately shows that it decreased more in GRID states than non-GRID states (7.5 vs. 3.4 percentage points). **Error! Reference source not found.** and Figure 13 show the correlation (y vs. x) between the difference in MVC campaign coverage (as depicted in Figure 10

and Figure 11) and the difference in target population (as depicted in **Error! Reference source not found.**) from which no trend can be discerned. We regressed these differences against each other in Model 4a (See Annex A for details of the model and Annex F for the model outputs) and indeed found no correlation. Similarly, no correlation was found with zero-dose coverage (Model 4b)

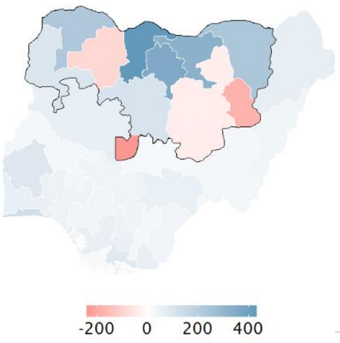

Figure 9 - Difference in the estimated target population between the 2016 and 2018 MVC in thousands

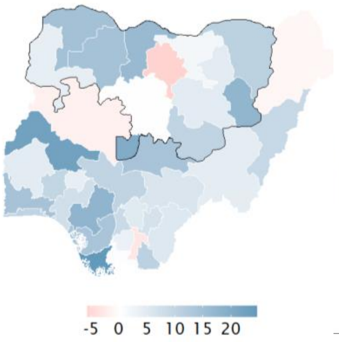

Figure 10 - Difference in the post-campaign immunisation coverage between the 2016 and 2018 MVC in percentage points

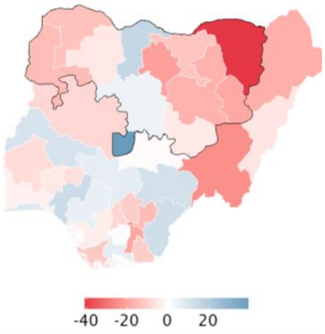

Figure 11 - Difference in the post-campaign zero-dose coverage between the 2016 and 2018 MVC

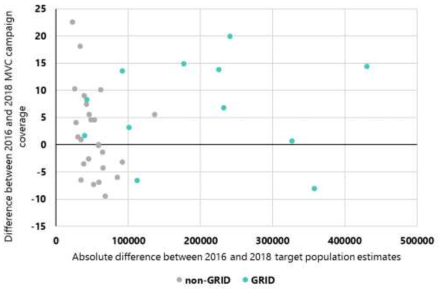

Figure 12 - Scatterplot of the difference between the 2016 and 2018 MVC campaign coverage (Y) and the absolute difference between the 2016 and 2018 target population estimates (x)

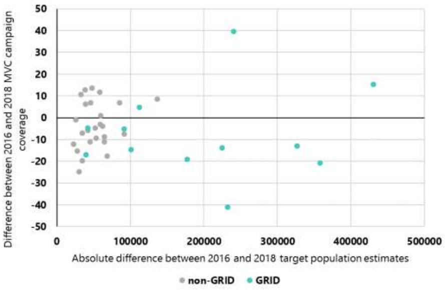

Figure 13 - Scatterplot of the difference between the 2016 and 2018 MVC zero-dose coverage (Y) and the absolute difference between the 2016 and 2018 target population estimates (x)

Discussion

Overall, our analyses <u>do not</u> provide conclusive evidence with regards to an effect of GRID on campaign coverage in the two instances examined. While we are unable to show an effect, this does not necessarily mean there is not effect – it simply means that, given the data available for analyses it is not possible to tell either way. Our overall finding is therefore that limitations with the data available in Nigeria mean that we cannot credibly show using quantitative methods whether, or not, GRID has made a difference. The main limitation with the data is that the metrics we used (post campaign coverage estimates) were both statistically and

‘epidemiologically’[5] underpowered. Moreover, we did not have all the necessary counterfactual information to estimate the effect of the geographic component on immunization coverage (i.e., number of settlements in the micro plans prior to the digital mapping and in the control areas).

While we see overall positive developments with regards to campaign coverage in Nigeria for both measles and polio, we cannot attribute these to more accurate population estimates and more precise maps. Indeed, measles SIA coverage improved slightly more in GRID supported states compared to non-GRID supported states, but we were not able to correlate these improvements to with changes in target population estimates. Similarly, there have been notable decreases between 2012 and 2019 in the number of children missed by polio SIA, but we were not able to conclusively attribute these to microplanning and VTS tracking.

Moreover, when interpreting the analyses presented here, it is important to bear in mind that there are two main components of the GRID approach to supporting vaccination campaigns, and we were only able to measure the effect of one at the time: the demographic component for measles campaigns, and the geographical component for polio. The *geographic component* which includes precise and complete maps and the *demographic component* consisting of more accurate population estimates. Both components are hypothesized to lead to better resource allocation and improved geographical coverage. The polio analyses were only able to partly assess the effect of the *geographic component*, and while we were unable to assess the effect of a demographic component, it was arguably not central to the polio intervention (see Annex G for details). Conversely, the measles microplans attempted to leverage both the *geographic and demographic component* of GRID, but whatever effect the *geographic component* may have on improving campaign outcomes, we could not estimate it for lack of a counterfactual (temporal or spatial): we do not know how many settlements were in the micro plans prior to the digital mapping, nor in the control areas.

One of the reasons we are unable to show an effect of GRID may be that our analyses are underpowered, both statistically (for measles) and epidemiologically (for both measles and polio). Lack of power means either of two things: a) there may be an effect and we can detect and b) maybe there is not effect. The measles analyses we performed state-level are statistically underpowered due to the small sample size available for analyses. Indeed, due to data availability (PMCCS campaign coverage estimates) these analyses could only be performed at state level meaning we have only 11 data points for analyses. With so few data points, the effect would have had to be a lot larger than what we observed (0.8 percentage points) to be able to reach statistical significance. More specifically, with the sample size available to us the difference between the average change in 2016 and 2018 MVC immunisation coverage in states with and without GRID should have been approximately 9 percentage points - which is more than 10 times the effect size observed. The polio analyses were adequately powered statistically since we had LGA level data for all campaigns conducted in Nigeria from 2012 to 2019, yet these were also not able to pick-up an effect of the intervention. But the lack of effect in both measles and polio analyses might be explained by a lack of epidemiological power. Indeed, the sampling frames used to collect the PMCCS and LQAS estimates were based on the census and by design excludes new settlements identified by GRID where most of the effects are more likely to have happened.

These limitations call for a reconsideration of the primary main metric used to assess the effect of GRID and suggest that immunization coverage may not be the right one, as it is both biased and removed from the program’s area of influence. The fact that we see a correlation between

5 The sampling frame for both surveys is based on the census enumeration areas, which does not include the areas added in the microplans by the improved mapping component of GRID – where one expects to find most of the benefits of the intervention in term of vaccination coverage. In that sense the analyses conducted on them can be defined as ‘epidemiologically under-powered’: they are not able to capture the entirety of an effect even if there is one because of the limitations in sampling frame’s geographical coverage5 .

improved geocooverage and improvements in polio SIA coverage suggests that GRID does have the *potential* to increase vaccination coverage, but we are simply not able to detect – perhaps because we were not able to assess changes in the 'newly found' areas (low epidemiological power). Our analyses were conducted exclusively on available data (i.e., secondary analyses of existing data), these relate only to immunization coverage data, and there are many as yet unverified assumptions between the GRID inputs and this ultimate impact indicator. Other campaign outcomes such as the total number of doses distributed, vaccine wastage and vaccine shortages are a more direct result of better resource allocation and campaign planning following GRID support. However, to the best of our knowledge, high quality data for these is not available digitally for analyses at the moment.

High quality vaccine distribution data (including wastage and shortage) could be very useful metric to analyses moving forward, but analyses of these data need to be contextualized within the wider process of actual use GRID outputs for program planning. Vaccine distribution, wastage and shortage data is very sensitive (especially in a context such as Nigeria where population estimates are very politically and economically charged) and thus prospective data collection directly from local health planning areas may be the best option to ensure high-quality unbiased data. Apart from providing very direct information about the use of GRID outputs for planning, this can also provide clues as to the accuracy of the new maps and population estimates. Indeed, one of the fundamental questions that remains open given our inability to detect and effect of GRID, regards the accuracy of the GRID outputs: are they actually better than the existing ones? The analysis of shortages and wastage data at local level could provide some insights: if new maps/population estimates *over*estimate actual population, we would expect a shortage of vaccines, and the other way round, if the new maps/population estimates *under*estimate actual population we would expect vaccine wastage. Ideally this should be accompanied by 1) data collection in a counterfactual area where GRID outputs are not used and 2) collection of information from actors at local level who are responsible for planning to understand how they use the GRID outputs and how it changes they modus operandi.

Alba S, *et al. BMJ Global Health* 2023; 8:e010938. doi: 10.1136/bmjgh-2022-010938

ANNEX A: Statistical models

Model 1 $Y_t = \beta_0 + \beta_1 M + \beta_2 X_t + \beta_3 MX_t + \beta_4 G + \beta_5 GM + \beta_6 GX_t + \beta_7 GXM_t T$

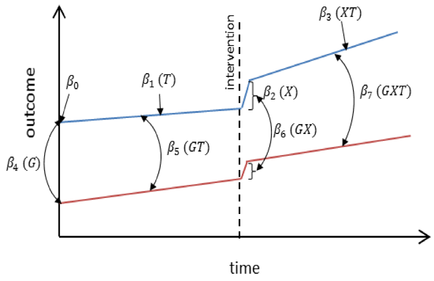

Interrupted time series linear regression model: In order to estimate the effect GRID on polio vaccination rates we fitted an interrupted time series regression model as detailed above and further described by Bernal et al[6] . Y_t represents the number of missed children at time t for a given LGA, M is a variable representing the number of months since January 2012 (the start of the LQAS time series) and X is a dummy variable indicating the period before and after 2015 (when GRID was scaled-up to the whole country) G represents the intervention group ($G = 1$) or control group ($G = 0$). G is time dependent, because LGAs sampled by LQAS are not constantly supported by GRID.

Where β_0 represents the number of missed children in Jan 2012 (M=0) β_1 is the change in number of missed children associated per time unit increase pre 2015 (representing the underlying pre-intervention trend), β_2 is the level change after 2015 in the non GRID-supported LGAs and β_3 indicates the slope change following 2015 (using the interaction between time and intervention: MX_t) β_4 represents the difference in GRID-supported LGAs at M=0, β_5 represents the slope difference between the GRID-supported and regular LGAs in the pre-intervention period, β_6 represents the difference between the change in level in the GRID-supported and non-GRID supported associated with the 2015, β_7 represents the difference between the change in slope in the GRID-supported and non-GRID supported associated with GRID.

The logic behind the model is as follows: We expect a steeper decrease in the number of missed children in the GRID-supported LGAs both before 2015 (when GRID was used in the northern states) and after 2015 (when GRID was upscaled to other states). Therefore β_5 and β_7 are the parameters of interest for the measures of effect of GRID.

6 https://academic.oup.com/ije/article/47/6/2082/5049576

Model 2 (a) $Y1_t = \beta_0 + \beta_1 M'_t\,; Y2_t = \beta_0 + \beta_1 M'_t$
(b) $Y_t = \beta_0 + \beta_1 S_t + \beta_2 X1_t + \beta_2 X1_t$

Linear regression model: In order to estimate trends over time in geocoverage as well as the relationship between immunisation coverage and geocoverage we fitted two types linear regression models at LGA level. Model (a) was used to estimate trends over time geocoverage statistics over time for campaigns that were tracked with the VTS, where $Y1_t$ represents the indicator of geocoverage 'proportion of settlements visited per LGA' at time t and $Y2_t$ indicates 'average geocoverage of visited settlements per LGA' at time t; M' is a variable representing the number of months since the first campaign tracking in the LGA. Model (b) is an LGA level model used to estimate the relationship between immunisation coverage and geocoverage indicators where Y_t denotes the number of children missed according LQAS surveys, S denotes the yearly secular trend (taking values between 2012-2019), $X1_t$ represents the indicator of geocoverage 'proportion of settlements visited per LGA' at time t and $X2_t$ indicates 'average geocoverage of visited settlements per LGA' at time t. These models were fitted using the Stata commend meglm with nested random intercept effects for states and LGA to account for clustering.

The model of primary interest for attribution is Model (b). The logic behind this model is as follows: if changes can be attributed to GRID, we would LGAs with fewer missed children by LQAS (=higher immunization coverage) to have higher geocoverage indicators. Thus is the parameter of interest in these analyses are beta2 and beta3 and we hypothesise that they should take a negative value to provide evidence that GRID is contributing to higher immunization coverage.

Model 3 $\gamma = \beta 0 + \beta 1 * [GRID] + \beta 2 * [\Delta Target] + \beta 3 * [GRID * \Delta Target]$

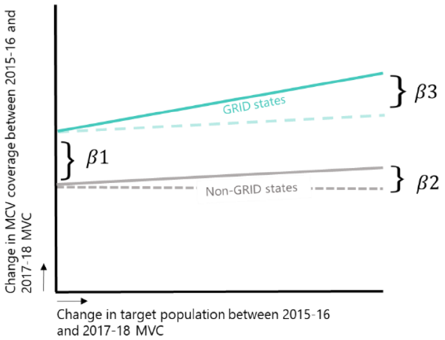

Linear regression model. Were γ is the outcome variable: difference in overall campaign vaccination coverage (model a) or zero-dose coverage (model b) between 2015-16 and 2017-18 campaign at state-level. $\beta 0$ is the difference in campaign coverage of 2015-16 versus 2017-18 in non-GRID states. $\beta 1$ is the difference between the difference in campaign vaccination coverage between GRID and non-GRID states. $\beta 2$ is the change in the difference between 2015-16 and 2017-18 campaign coverage for every 10,000 increase in the difference between 2015-16 and 2017-18 target population. $\beta 3$ is the difference between GRID and non-GRID states in the change in the difference in campaign coverage for every 10,000 increase in the difference in target population.

The logic behind this model is as follows: if changes to vaccination coverage can be attributed to GRID, we would expect states with larger changes in population target to have achieved larger increases in vaccination coverage, while at the same time not observing such an association in the non-GRID states. $\beta 3$ can inform us on this differential effect and as such is is the parameter of interest to estimate the effect of GRID.

Model 4 $logit(P(\gamma = 1) = \beta0 + \beta1 * [Time] + \beta2 * [GRID] + \beta3 * [Time * GRID]$

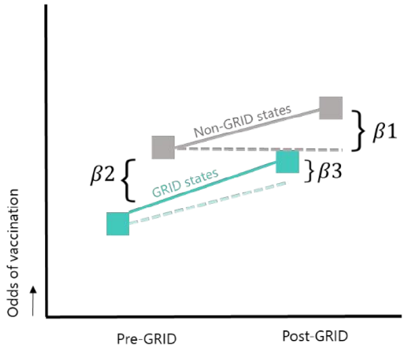

Logistic regression model. Were γ is the outcome variable: children aged 9-59 months who received MCV during the campaign ($\gamma = 1$, model a) or children aged 9-59 months who received MCV for the first time during the campaign ($\gamma = 1$, model b). Exp($\beta1$) is the odds ratio of the odds being vaccinated in 2017-18 over the odds of being vaccinated in 2015-16, in non-GRID states. Exp($\beta2$) is the odds ratio of being vaccinated in GRID states versus non-GRID states during the 2016 campaign.Exp($\beta3$) the difference between odds ratio of being vaccinated in 2016 versus 2018 in GRID states and the odds ratio of being vaccinated in 2016 versus 2018 in non-GRID states.. The model was adjusted for the clustered sampling design using the package 'survey' in R. The *svyglm* model returns 'model-robust' standard errors

The logic behind this model is as follows: if changes can be attributed to GRID, we would expect a larger increase over time in the GRID states compared to the non-GRID states. Beta3 can inform us on this differential effect and as such is the parameter of interest to estimate the effect of GRID.

ANNEX B: TECHNICAL NOTES ON LQAS AND LINK BETWEEN LQAS and VTS

Polio immunisation coverage was assessed between 2012 and 2019 by means of lot quality assurance surveys (LQAS). LQAS have been shown to be useful and a statistically reliable tools for monitoring polio vaccination campaign quality7. From and operational perspective, it helps identify areas with high or low coverage quality. For monitoring purposes, it enables to track trends in campaign quality over time. The LQAS methodology has been developed and piloted tested for Nigeria specifically with the following characteristics since 2012: 1) One lot of 60 children is selected per LGA comprising of 6 clusters of 10 children each; 2) Six wards are selected per LGA, using probability proportional to size (PPS), and 1 settlement per ward; 3) the random selection of settlements is performed using a master list of settlements, rather than wards, so all settlements in an LGA stand an equal chance of being selected; 4) new framework for lots of 60 children is as follows: 0–3 unvaccinated: "accepted at 90%"; 4–8 unvaccinated: "accepted at 80%"; 9+ = "not accepted at 80%" An additional threshold of "accepted at 60%" and "not accepted at 60%," with an associated d value of 20+ unvaccinated children out of 60, was adapted to differentiate between areas of particularly weak coverage. **The outcome variable in our analysis was the number of unvaccinated (missed) children per LGA as a numerical value between 1 and 60 rather than in this above mentioned categories, in order to make the most out of the data collected in the LQAS.**

While LQAS are conducted after each polio immunization campaign, the VTS only has campaign data for states/LGAs that are tracked. Each year since 2012, BMGF has supported tracking in a set number of LGAs (which are selected by the national Polio EOC) for each campaign. The maximum was 80 LGAs/campaign and that number has been gradually dropping over the past 5 years. In other words, the VTS only tracks campaigns a number of LGAs in any given round.

VTS tracking was implemented in nine Northern states between 2012 and 2015 and in the remaining states thereafter – although not all states were covered across all years (**Error! Reference source not found.** table). Thirteen out of 36 states were not covered by VTS throughout the 2012-2019 period: Abia, Akwa Ibom, Bayelsa, Benue, Cross River, Delta, Ekiti, Imo, Nasarawa, Ondo, Plateau, Rivers. Kaduna and Bauchi were mapped shortly after the 8 initial states (Kebbi, Zamfara, Sokoto, Katsina, Kano, Jigawa, Yobe, Borno), but there were some security issues in Kaduna and the mapping was never fully completed. While Kaduna participated in all the campaigns that were conducted in the North, the Kaduna polio EOC also declined all requests for tracking due to the security issues until 2019.

Year	States
2012	Jigawa, Kano, Sokoto, Zamfara, Katsina,
2013	Jigawa, Kano, Sokoto, Zamfara, Katsina, Kebbi, Borno
2014	Jigawa, Kano, Sokoto, Zamfara, Katsina, Kebbi, Bauchi
2015	Jigawa, Kano, Sokoto, Zamfara, Katsina, Kebbi, Bauchi, Yobe
2016	Jigawa, Kano, Sokoto, Zamfara, Katsina, Kebbi, Bauchi, Yobe, Adamawa Borno Gombe Taraba
2017	Jigawa, Sokoto, Zamfara, Katsina,, Bauchi, Yobe, Adamawa Borno, Kaduna
2018	Adamawa Borno Kaduna Sokoto Yobe Ebony Gombe Jigawa Katsina Bauchi Taraba Zamfara Kano
2019	Adamawa Bauchi Borno FCT Kaduna Kwara Oyo Kaduna Kebbi Kwara Niger Sokoto Zamfara Lagos Ogun Kogi Osun Anambra Edo Enugu

7 https://academic.oup.com/jid/article/210/suppl_1/S333/2194124

ANNEX C: TECHNICAL NOTES ON PMCCS

The 2015-16 MVC was implemented in two parts, starting with 19 Northern states in November 2015 and followed by 17 Southern states in January 2016. In this round of MVC none of the states had implemented GRID as part of their microplanning process. The PMCCS, which is planned to be conducted directly after the MVC, was conducted in January 2016 for the Northern states and in February 2016 in the Southern states. Data from this PMCCS is used as a baseline for Model 3 (pre-GRID odds of being vaccinated) and Model 4 (pre-GRID vaccination campaign coverage)

The process of microplanning using GRID started in April 2017 in preparation for the 2017-18 MVC. Alike the previous MVC, the 2017-18 MVC was first implemented in October first in the Northern states followed by the Southern states in February 2018. The PMCCS 2018 were conducted in the Northern states in January 2018 and in the Southern states in April 2018. This dataset is used as the endline for Model 3 (post-GRID vaccination campaign coverage) and Model 4 (post-GRID odds of being vaccinated).

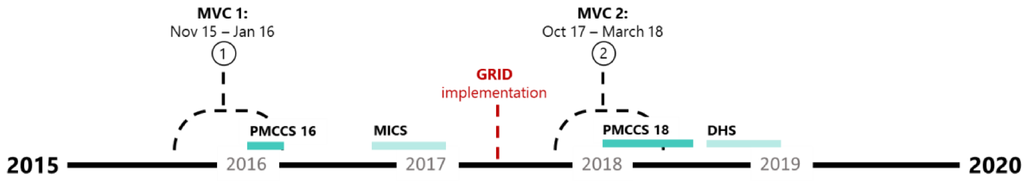

Figure 14 - Overview of implementation of Measles Vaccination Campaigns (MVC), Post Measles Campaign Coverage Surveys (PMCCS), Demographic and Health Survey (DHS), Multiple Indicator and Cluster Survey (MICS) and GRID

ANNEX D: LQAS coverage estimates by LGA and by year

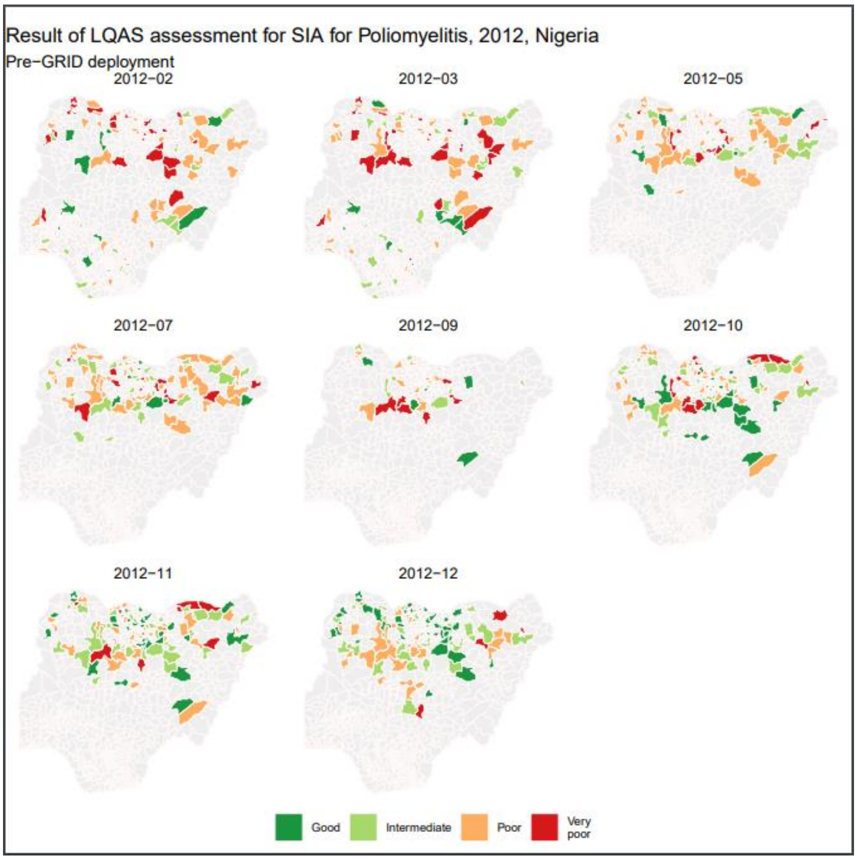

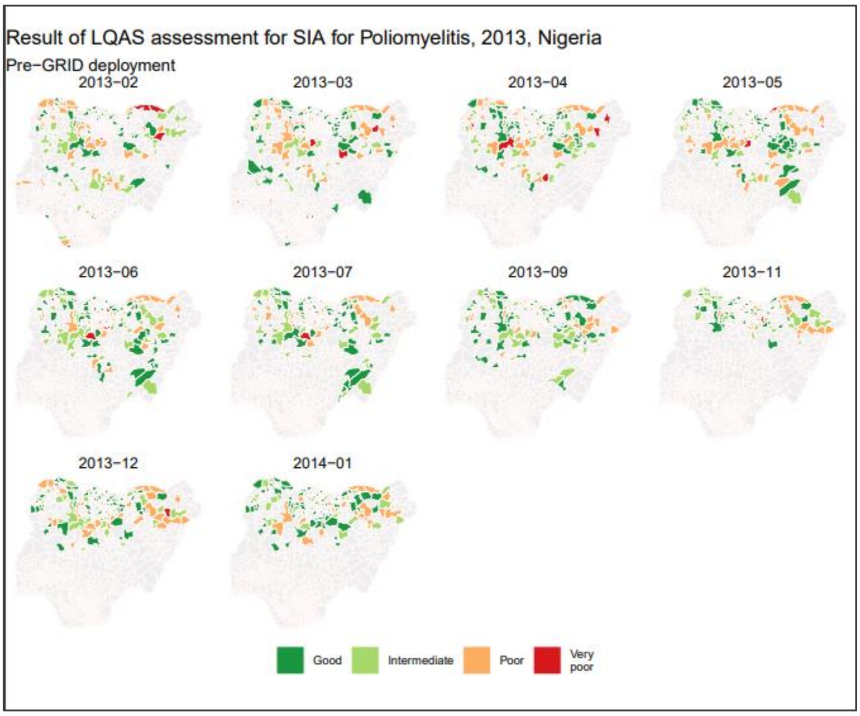

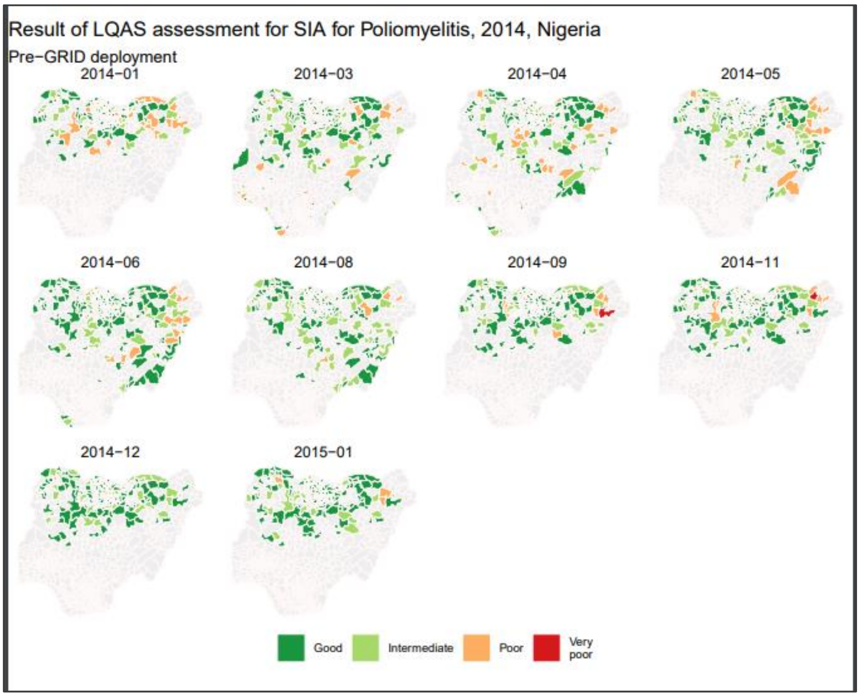

Alba S, *et al. BMJ Global Health* 2023; 8:e010938. doi: 10.1136/bmjgh-2022-010938

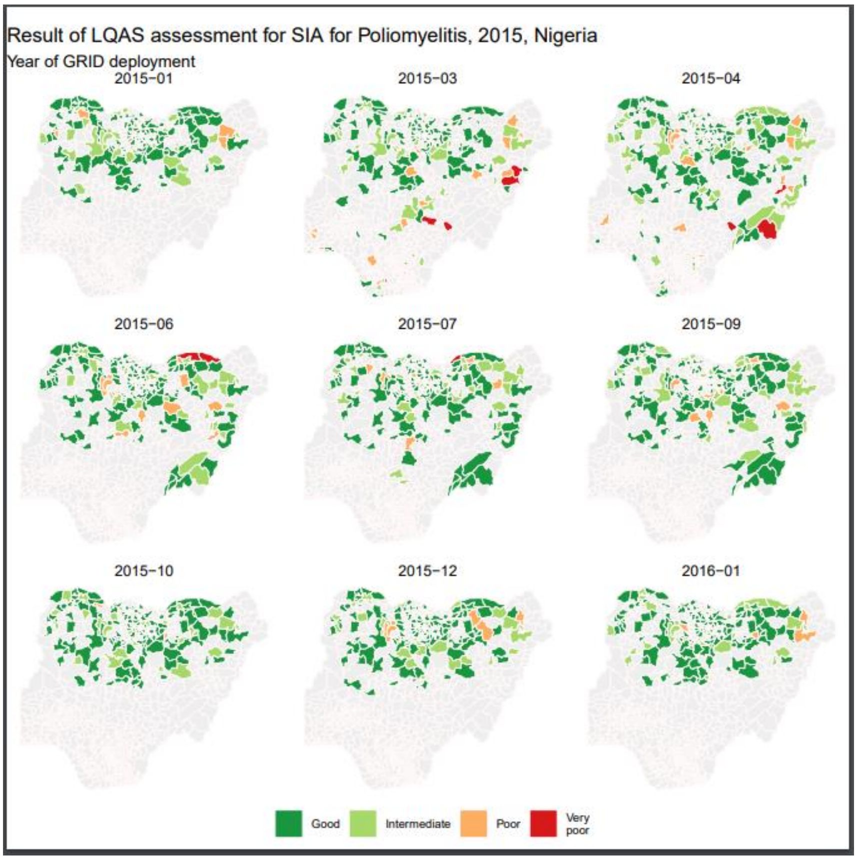
Result of LQAS assessment for SIA for Poliomyelitis, 2015, Nigeria
Year of GRID deployment
2015-01
2015-03
2015-04
2015-06
2015-07
2015-09
2015-10
2015-12
2016-01
Good
Intermediate
Poor
Very poor

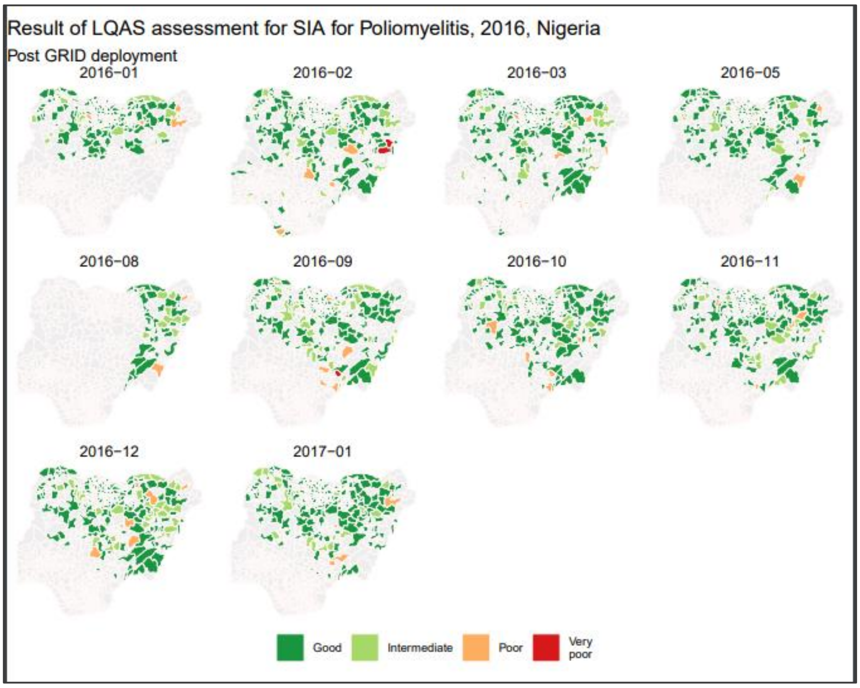
Result of LQAS assessment for SIA for Poliomyelitis, 2016, Nigeria
Post GRID deployment
2016-01
2016-02
2016-03
2016-05
2016-08
2016-09
2016-10
2016-11
2016-12
2017-01
Good
Intermediate
Poor
Very poor

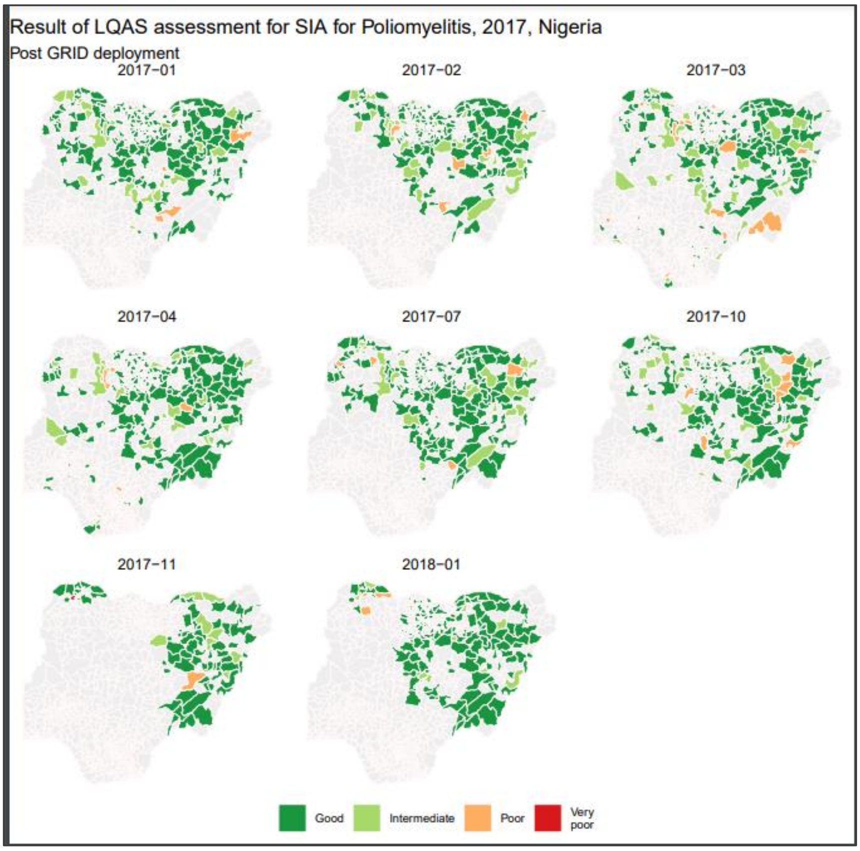
Result of LQAS assessment for SIA for Poliomyelitis, 2017, Nigeria
Post GRID deployment
2017−01
2017−02
2017−03
2017−04
2017−07
2017−10
2017−11
2018−01
Good
Intermediate
Poor
Very poor

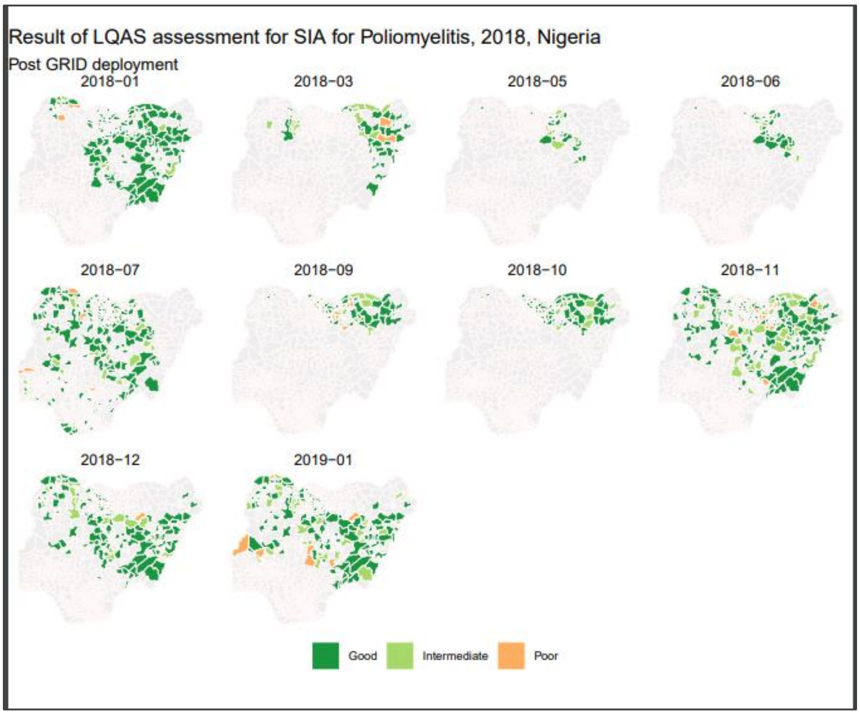
Result of LQAS assessment for SIA for Poliomyelitis, 2018, Nigeria
Post GRID deployment
2018−01
2018−03
2018−05
2018−06
2018−07
2018−09
2018−10
2018−11
2018−12
2019−01
Good
Intermediate
Poor

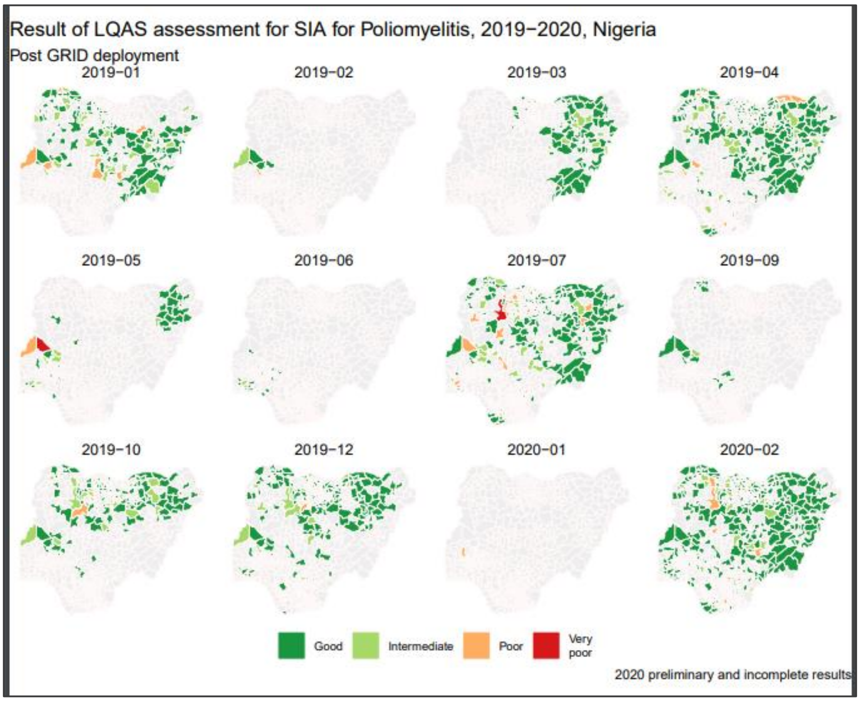
Result of LQAS assessment for SIA for Poliomyelitis, 2019−2020, Nigeria
Post GRID deployment
2019−01
2019−02
2019−03
2019−04
2019−05
2019−06
2019−07
2019−09
2019−10
2019−12
2020−01
2020−02
Good
Intermediate
Poor
Very poor
2020 preliminary and incomplete results

Year	Campaign	Coverage (States)	Coverage (#LGAs)
2012	Oct 2012 IPD (2012-10-06-2012-10-10	Jigawa, Kano	6
	Nov 2012 IPD (2012-11-17-2012-11-21)	Jigawa, Kano, Sokoto, Zamfara	9
	Dec 2012 IPD (2012-12-15-2012-12-19)	Jigawa, Kano, Katsina, Sokoto, Zamfara	10
2013	Jan 2013 IPD (2013-01-15-2013-01-19)	Sokoto	4
	Feb 2013 IPD (2013-02-03-2013-02-08)	Kano	16
	Mar 2013 IPD (2013-03-02-2013-03-07)	Katsina, Sokoto, Zamfara	24
	Apr 2013 IPD (2013-04-13-2013-04-18)	Jigawa, Kano, Katsina, Sokoto, Zamfara	28
	Apr 2013 IPD (FCT) (2013-04-13-2013-04-18)	N/A	N/A
	Jun 2013 IPD (2013-06-15-2013-06-21)	Kano Katsina, Kebbi, Sokoto, Zamfara	21
	Jul 2013 IPD (2013-07-06-2013-07-11)	Kano Katsina, Kebbi, Sokoto, Zamfara	24
	Sep 2013 IPD (2013-10-27-2013-09-16)	Kano Katsina, Sokoto, Zamfara	40
	Oct 2013 IPD (2013-10-27-2013-10-31)	Kano	6
	Nov 2013 IPD (2013-11-16-2013-11-25)	Borno Kano Katsina, Sokoto, Zamfara	40
	Dec 2013 IPD (2013-12-14-2013-12-23)	Jigawa, Kano, Katsina, Sokoto, Zamfara	41
2014	Jan 2014 IPD (2014-01-25-2014-02-03)	Jigawa, Kano, Katsina, Sokoto, Zamfara	40
	Mar 2014 IPD (2014-03-01-2014-03-07)	Kano	16
	Mar 2014 Mop-up (Kano) (2014-03-22-2014-03-26)	Kano, Katsina, Sokoto, Zamfara	37
	Apr 2014 IPD (2014-04-11-2014-04-17)	Bauchi, Kano, Katsina, Sokoto, Zamfara	60
	April 2014 Mop-up (Kano) (2014-05-01-2014-05-04)	Kano	4
	May 2014 IPD (2014-05-24-2014-05-30)	Kano, Katsina, Sokoto,	60
	Jun 2014 IPD (2014-08-09-2014-08-14)	Bauchi Jigawa Kano Katsina Sokoto	60
	Aug 2014 IPD (2014-08-09-2014-08-14)	Jigawa Kano Katsina Sokoto	58

	Aug 2014 Mop-up (Kano) (2014-08-31-2014-09-04)	Kano	8
	Sep 2014 IPD (2014-09-18-2014-09-26)	Bauchi Kano Katsina Sokoto Zamfara	60
	Oct 2014 Mop-up (Kano) (2014-10-11-2014-10-16)	Kano	8
	Nov 2014 IPD (2014-11-01-2014-11-07)	Bauchi Kano Katsina Kebbi Sokoto	80
	Dec 2014 IPD (2014-12-11-2014-12-17)	Bauchi Kano Katsina Kebbi Sokoto Zamfara	80
2015	Jan 2015 IPD (2015-01-22-2015-01-28)	Bauchi Jigawa Kano Katsina Kebbi Sokoto Zamfara	80
	Mar 2015 IPD (2015-03-12-2015-03-19)	Bauchi Jigawa Kano Katsina Kebbi Sokoto Zamfara	80
	Apr 2015 IPD (2015-06-06-2015-05-01)	Bauchi Jigawa Kano Katsina Kebbi Sokoto Zamfara	80
	Jun 2015 IPD (2015-06-06-2015-06-11)	Bauchi Jigawa Kano Katsina Sokoto Zamfara	80
	Jul 2015 IPD (2015-07-25-2015-07-31)	Bauchi Jigawa Kano Katsina Kebbi Sokoto Zamfara	80
	Sep 2015 IPD (2015-09-05-2015-09-10)	Bauchi Jigawa Kano Katsina Kebbi Sokoto Zamfara	80
	Oct 2015 IPD (2015-10-15-2015-10-22)	Bauchi Jigawa Kano Katsina Kebbi Sokoto Yobe Zamfara	80
	Dec 2015 IPD (2015-12-03-2015-12-10)	Bauchi Jigawa Kano Katsina Kebbi Sokoto Yobe Zamfara	80
2016	Jan 2016 IPD (2016-01-21-2016-01-27)	Bauchi Jigawa Kano Katsina Kebbi Sokoto Yobe Zamfara	80
	Feb 2016 IPD (2016-02-27-2016-03-07)	Benue FCT Gombe Kogi Kwara Niger Plateau Taraba	27
	Mar 2016 IPD (2016-03-19-2016-03-24)	Bayela Cross-River Delta Edo Ekiti Lagos Ogun Ondo Osun Oyo	26
	May 2016 IPD (2016-05-12-2016-05-18)	Bauchi Katsina Yobe	24
	Aug 2016 IPD (2016-08-27-2016-09-02)	Adamawa Borno Gombe Taraba Yobe	37
	Sep 2016 IPD (2016-09-17-2016-09-22)	Adamawa Kigawa Kano Katsina Yobe	35
	Oct 2016 IPD (2016-10-15-2016-10-25)	Adamawa Borno FCT Gombe Taraba Yobe	49
	Nov 2016 IPD (2016-11-12-2016-11-18)	Adamawa Benue Borno Gombe Taraba Yobe	46
	Dec 2016 IPD (2016-12-01-2016-12-07)	N/A	N/A
	Dec 2016 IPD Phase II (2016-12-16-2016-12-26)	Borno Sokoto	2
2017	Jan 2017 IPD (2017-01-28-2017-02-05)	Bauchi Borno Sokoto Yobe Zamfara	41

	Feb 2017 IPD (2017-02-23-2017-03-08)	Adamawa, Bauchi Borno Jigawa Kano Katsina Sokoto Yobe	66
	Mar 2017 IPD (2017-03-23-2017-03-30)	Adamawa, Bauchi Borno Jigawa Sokoto Yobe Zamfara	58
	Apr 2017 IPD (2017-04-20-2017-04-29)	Adamawa, Bauchi Borno Yobe	42
	May 2017 Zamfara IPD (2017-05-13-2017-05-17)	Zamfara	5
	May 2017 Sokoto IPD Phase 1 (2017-05-20-2017-05-26)	Sokoto	5
	May 2017 Sokoto IPD Phase 2 (2017-05-27-2017-05-31)	Sokoto	18
	Jul 2017 IPD (2017-07-08-2017-07-16)	Borno Sokoto Yobe	55
	Microplan Tracking for Kaduna and IPD for Sokoto (2017-08-14-2017-08-27)	Kaduna Sokoto	46
	Microplan Tracking for Kaduna and Sokoto (2017-09-04-2017-09-17)	Sokoto and Kaduna	46
	Microplan Tracking for Sokoto (2017-09-25-2017-10-01)	Sokoto	23
	Oct 2017 IPD Phase 1 (2017-10-04-2017-10-11)	Adamawa, Borno	10
	Oct 2017 IPD Phase 2 (2017-10-11-2017-10-22)	Borno Kaduna Sokoto Yobe	48
	Nov 2017 IPD (2017-11-02-2017-11-15)	Adamawa bauch Borno Sokoto Yobe	63
	Demo VTS Campaign definition (2017-11-27-2017-11-29)		2
2018	VTS Campaign Dry run (2018-01-10-2018-01-12)	Borno Kano	4
	Jan 2018 IPD Campaign (2018-01-20-2018-01-25)	Adamawa Borno Kaduna Sokoto Yobe	58
	Mar 2018 IPD Campaign (2018-03-02-2018-03-08)	Adamawa Borno Yobe	31
	Apr 2018 IPD Campaign (2018-04-06-2018-04-12)	Adamawa Borno Jigawa Yobe	34
	May 2018 OBR Campaign (2018-05-10-2018-05-15)	Gombe Jigawa Sokoto	36
	May 2018 OBR Campaign Phase 2 (2018-05-26-2018-06-01)	Gombe Jigawa Sokoto	36
	Jun 2018 IPD Campaign (2018-06-30-2018-07-04)	Ebony Gombe Jigawa Sokoto	32
	Jul 2018 IPD Campaign Borno (2018-07-14-2018-07-18)	Borno	11
	Sep 2018 OBR Campaign (2018-09-01-2018-09-09)	Borno Jigawa Katsina Sokoto Yobe	47

	Oct 2018 OBR Campaign (2018-10-06-2018-10-12)	Bauchi Borno Jigawa Kano Katsina Sokoto Yobe	92
	Nov 2018 IPD Campaign (2018-11-03-2018-11-08)	Adamawa Borno	17
	Dec 2018 OBR Campaign (2018-12-08-2018-12-18)	Adamawa, Bauchi Borno, Gombe Kaduna Kano Katsina Taraba Zamfara	48
2019	Jan 2019 OBR Campaing (2019-01-23-2019-01-31)	Adamawa Bauchi Borno FCT Kaduna Kwara Oyo	48
	Feb 2019 OBR Campaign (2019-02-09-2019-02-13)	Kwara Oyo	10
	Mar 2019 IPD Campaign (2019-03-16-2019-03-22)	Borno	22
	Apr 2019 OBR Campaign (2019-04-13-2019-04-17)	Kaduna Kebbi Kwara Niger Sokoto Zamfara	42
	Apr 2019 Phase 2 OBR Campaign (2019-04-27-2019-05-03)	Borno Jigawa Kano Yobe	10
	April 2019 Phase 3 OBR Campaign (2019-05-04-2019-05-08)	Adamawa Katsina	10
	May 2019 OBR Phase 1 Campaign (2019-05-08-2019-05-23)	Lagos Ogun Oyo	12
	May 2019 OBR Phase 2 Campaign (2019-05-25-2019-05-30)	Borno	23
	Jun 2019 OBR Phase 1 Campaign (2019-06-14-2019-06-24)	Ogun Oyo	12
	Jul 2019 IPV/OBV Phase 1 Campaign (2019-08-13-2019-08-14)	Borno	23
	Sep 2019 OBR Campaign (2019-09-14-2019-09-24)	Kogi Kwara Osun Oyo Sokoto	48
	Oct 2019 OBR/SIPD Campaign (2019-10-12-2019-10-23)	Anambra Borno Edo Enugu Kogi	43
	Nov 2019 IPD Campaign (2019-11-02-2019-11-06)	Sokoto	10
	Dec 2019 OBR Campaign (2019-12-07-2019-12-16)	Borno Kogi	2

ANNEX E: Outputs for Model 1 and Model 2 (Polio)

Model 2

The interrupted time series model (See description of Model 1 in Annex A) reported in the table below provides quantifications of the trends in the estimated average number of children missed by Polio SIA (according to LQAS) in Phase 1 (2012-2015) and Phase 2 (2016-2019) of GRID implementation. The number of missed children significantly decreased by 0.30 per month before 2015 in the regular campaigns (beta1). On average, there were 8.15 significantly fewer missed children in the regular campaigns after 2015 compared to before (beta2) After 2015 the number of missed children started decreasing significantly less than before (0.04 cases per month which is derived by beta3+beta1=0.26-0.30) in regular campaigns. On average the GRID supported campaigns had 2.34 fewer missed children than the regular campaigns at baseline (beta4). The number of missed children pre-2015 decreased a slightly faster rate in the regular campaigns compared to the GRID-supported compared. The magnitude of this difference is negligible (0.07 children per month) - albeit statistically significant (beta5). Post 2015 there are no statistically significant differences in the number of children missed in both types of campaigns (beta6), nor in the slopes over time (beta7) As described in Annex A the coefficients of interest to evaluate the effect of GRID are beta5 and beta7. From the estimated effects of these coefficients we concluded that a slight effect of GRID was observed pre-2015 when the GRID was implemented in the northern states, where it appears to have contributed very slightly to a faster decrease in the number of missed children. However, a noticeable strong down-ward trend was already underway in the other states. Post-2015 there is not difference between the GRID supported and regular campaigns.

	beta	95%CI Lower bound	Upper bound	P-value
**Outcome variable: Number of missed children by LQAS**				
Time (month since Jan 2012) β_1	-0.30	-0.33	-0.26	0.0000
2015 (After vs. before) β_2	-8.15	-9.50	-6.80	0.0000
2015*Time β_3	0.26	0.22	0.30	0.0000
GRID supported β_4	-2.34	-3.74	-0.94	0.0010
**GRID supported*Time $\boldsymbol{\beta_5}$**	**0.07**	**0.01**	**0.13**	**0.0340**
GRID supported*2015 β_6	0.93	-1.22	3.08	0.3930
**GRID supported*Time*2015 $\boldsymbol{\beta_7}$**	**-0.04**	**-0.11**	**0.02**	**0.1990**

Alba S, *et al. BMJ Global Health* 2023; 8:e010938. doi: 10.1136/bmjgh-2022-010938

Model 2 (a)

	beta	95%CI<br>Lower bound	Upper bound	P-value
**Exposure variable: time in months since start of tracking (2012-2014)***				
Proportion of settlements visited				
β_0	72.92	65.68	80.16	<0.0001
β_1	0.47	0.40	0.54	<0.0001
Geocoverage of visited settlements				
β_0	63.06	54.79	71.33	<0.0001
β_1	0.61	0.53	0.70	<0.0001
**Exposure variable: time in months since start of tracking (2015-2019)***				
Proportion of settlements visited				
β_0	66.70	55.71	77.65	<0.0001
β_1	0.14	0.11	0.18	<0.0001
Geocoverage of visited settlements				
β_0	60.34	48.89	71.79	<0.0001
β_1	0.04	0.01	0.07	<0.0001

*Effect of time in Phase 1 (2012-2014) is statistically different from effect in Phase 2 (2015-2019) (as per p-value provided by interaction term between time in months and Phase)

Model 2 (b)

	beta	95%CI<br>Lower bound	Upper bound	P-value
**Outcome variable: number of missed children according to LQAS**				
Simple regression				
Year	-1.14	-1.19	-1.10	<0.0001
Proportion of settlements visited	-1.09	-2.40	0.23	0.105
Geocoverage of visited settlements	-0.04	-0.07	-0.01	0.019
Multiple regression				
Year	-0.61	-0.78	-0.45	<0.0001
Proportion of settlements visited	0.12	-1.17	1.41	0.858
Geooverage of visited settlements	-0.06	-0.08	-0.03	<0.0001

Alba S, *et al. BMJ Global Health* 2023; 8:e010938. doi: 10.1136/bmjgh-2022-010938

ANNEX F: Outputs for Model 4a and b, and Model 5a and b (Measles)

The logistic difference-in-difference model (see description of Model 3 in Annex A) reported in the table below provides quantifications of the trend in the odds of being immunized during the MCV before and after GRID implementation and between GRID and non-GRID states. The model was made for (a) children aged 9-59 irrespective of immunization status prior to the campaign and (b) children age 9-59 who were immunized for the first time during the campaign.

Children in GRID states were 29% less likely to be vaccinated during the 2016 MVC as compared to children in non-GRID states (OR: 0.71, 95%CI: 0.65 – 0.77). The odds of being vaccinated during the 2018 campaign as compared to the 2016 campaign increased by a factor of 1.29 in non-GRID states and by a factor of 1.70 in GRID states. This means that the increase in the odds of being vaccinated during 2018 campaign versus the 2016 campaign is 1.30 times higher in GRID areas as compared to non-GRID states. In other words campaign effectiveness increased across the country, but especially in GRID states.

The odds of being vaccinated for the first time during the 2016 MVC were 2.84 (95%CI: 2.64 – 3.06) times higher in GRID states as compared to non-GRID states. There is no apparent effect in first time vaccinations over time in both GRID and non- GRID states.

The results of these models suggest that while improved campaign coverage between the 2016 and 2018 MVC was achieved in GRID states over non-GRID states, most of these children had already been vaccinated prior to the campaign.

	OR	95%CI<br>Lower bound	Upper bound	P-value
**3a. Outcome variable: Children aged 9-59 months who received MCV during the last campaign**				
Constant	6.14	5.82	6.48	<0.0001
Time	1.29	1.18	1.43	<0.0001
Grid	0.71	0.65	0.77	<0.0001
Grid::Timeβ_3	1.30	1.12	1.52	0.001
**3b. Outcome variable: Children aged 9-59 months who received MCV for the first time during the last campaign**				
Constant	0.45	0.43	0.47	<0.0001
Time	0.95	0.89	1.03	0.207
Grid	2.84	2.64	3.06	<0.0001
Grid::Time	1.06	0.94	1.19	0.345

Time reflects the OR of receiving MCV (for first time) between the 2015-16 campaign and the 2017-18 campaign in non-GRID states. **GRID** reflects the OR of receiving MCV (for first time) during the 2015-16 campaign between GRID and non-GRID states. **Grid::Time** reflects the difference between GRID and non-GRID states between the OR of receiving MCV (for the first time) during the 2015-16 campaign and the 2017-18 campaign

The linear regression model (see description of Model 4 in Annex A) reported in the table below provides quantifications of the correlation between a change in target population between the 2016 and 2018 measles vaccination campaign (MVC) and a change in the post-campaign coverage between the 2016 and 2018 MVC among children aged 9-59 months (a) irrespective of immunization status prior to the campaign and (b) who were immunized for the first time during the campaign (zero-dose), on a state level.

For every 10,000 increase in the change in the estimated target population between the 2016 and the 2018 MVC, the difference in the post campaign coverage between the 2016 and 2018 campaign decreases with 0.70 percentage points in non-GRID states (beta1) and increases with 0.01 percentage in GRID states (beta1+beta3). In addition to not being statistically significant, the differences are also negligible.

A change in target population has no apparent effect on the change in zero-dose coverage. For every increase in the change in the estimated target population between the 2015-16 and 2017-18 campaign of 10,000, the zero-dose coverage increases with 1.04 percentage points in non-GRID states (beta1) and with 0.22 percentage points in GRID states (beta1+beta3).

	B	95%CI Lower bound	Upper bound	P-value
**4a. Outcome variable: Absolute difference in state-level MCV campaign coverage in 2015-16 and 2017-18**				
Constant	11.92	5.00	18.84	0.002
ΔTarget population β_1	-0.70	-1.89	0.49	0.259
Grid β_2	-3.11	-13.67	7.44	0.567
Grid:: ΔTarget population β_3	0.71	-0.53	1.95	0.270
**4b. Outcome variable: Absolute difference in state-level MCV zero-dose campaign coverage in 2015-16 and 2017-18**				
Constant	-8.90	-22.69	4.88	0.214
ΔTarget population β_1	1.04	-1.32	3.41	0.394
Grid β_2	-2.90	-23.93	18.13	0.788
Grid:: ΔTarget population β_3	-0.82	-3.29	1.64	0.517

Target population reflects the association between a change in 10,000 target population and a change in MCV coverage/zero-dose coverage in non-GRID areas. **GRID** reflects the difference in the change in MCV coverage/zero-dose coverage between GRID and non-GRID areas when there is no difference in target population. **GRID::Target population** reflects the difference in the association between a change in target population and a change in MCV/zero-dose coverage between GRID and non-GRID states.

Alba S, *et al. BMJ Global Health* 2023; 8:e010938. doi: 10.1136/bmjgh-2022-010938

ANNEX G: 2016 and 2019 GRID population estimates

Population estimates were made in 2016 for 10 northern states by Oak Ridge National Laboratory models in from 2016-2018 and in 2019 by WorldPop for the whole of Nigeria as shown in the figures below. The 2016 Oak Ridge National Laboratory estimates were generally higher than the census estimates for all states, whereas the 2019 estimates were generally lower than the census estimate (bar a few states were increases can be seen). The 2016 and 2019 estimates bear no similarity, which may also reflects the different modelling approaches used. We were not able to estimate the effect of population changes in the polio analyses since 1) there was no differential effects in intervention vs. control areas to attribute to populations changes and 2) population estimates were only included in polio SIA microplans in Phase 2, and as can be seen from the table in Annex D, tracking for this phase was spread out and not repeated multiple times in a given areas (as opposed to in Phase 1 where tracking was done systematically and intensively in the northern states) thereby diluting the power of any attribution analyses.

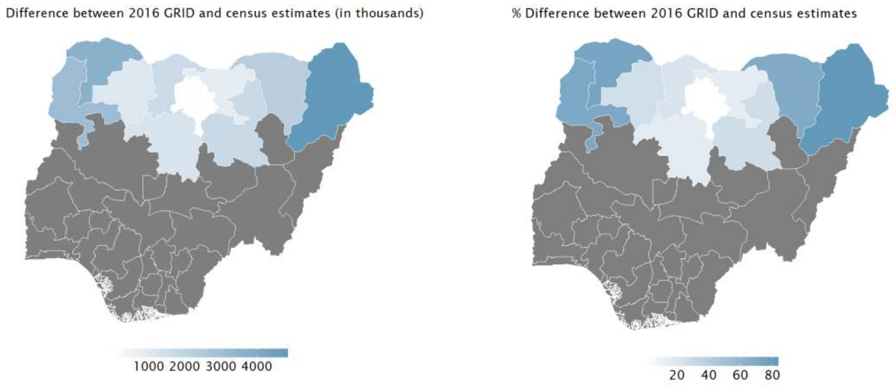

Difference between 2016 Oak Ridge National Laboratory population estimates and census projections

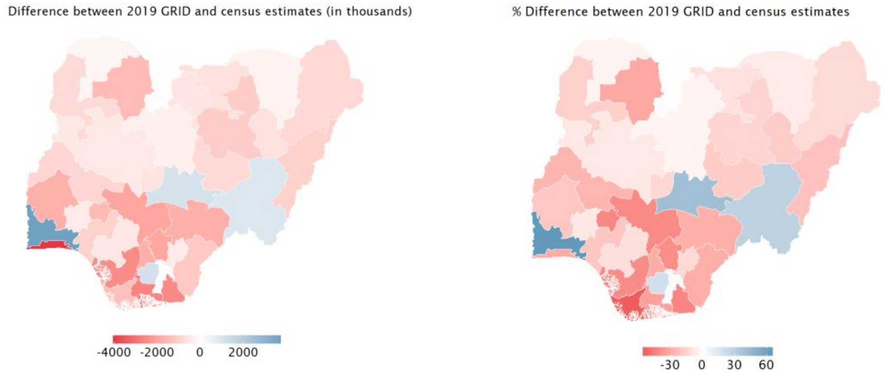

Difference between 2019 WorldPop GRID population estimates and census projections

KIT Royal Tropical Institute

itad

Itad is a global organisation. Our strategy, monitoring, evaluation and learning services work to make international development more effective. We generate evidence on important issues – from malnutrition to migration – to support our partners to make informed decisions and improve lives.

Itad.com @ItadLtd mail@itad.com

Itad Ltd
Preece House
Davigdor Road Hove,
East Sussex UK
BN3 1RE

+44 (0) 1273 765250

Itad Inc
c/o Open Gov Hub
1100 13th St NW, Suite 800
Washington, DC, 20005
United States

+1 (301) 814 1492
