# Supplementary figures and images for: Finagle’s laws of information: lessons learnt evaluating a complex health intervention in Nigeria
Source: BMJ Glob Health. 2023 Mar 24;8(3):e010938. doi: 10.1136/bmjgh-2022-010938 (PMC10040038; doi:10.1136/bmjgh-2022-010938)

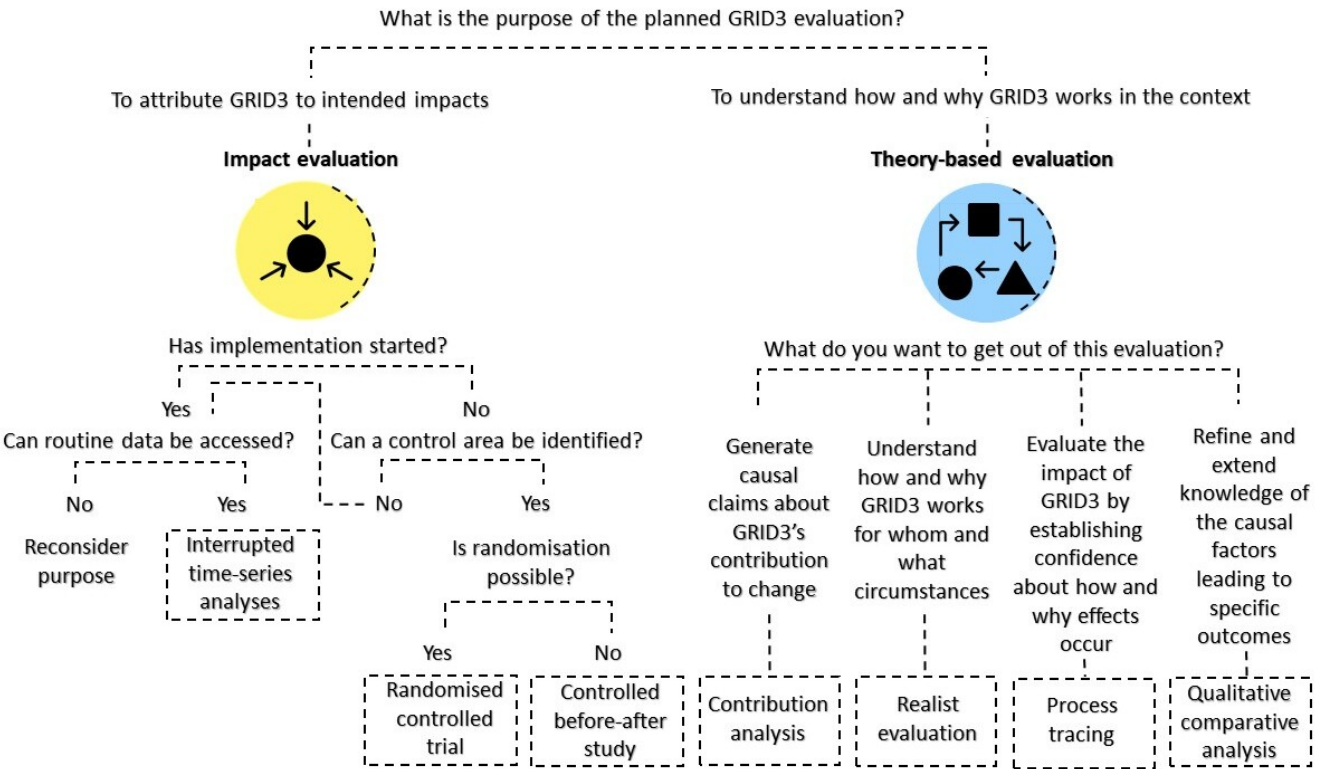

Supplement: Supplementary data [file bmjgh-2022-010938supp002.pdf]
